# Short-Chain Naphthoquinone Protects Against Both Acute and Spontaneous Chronic Murine Colitis by Alleviating Inflammatory Responses

**DOI:** 10.3389/fphar.2021.709973

**Published:** 2021-08-23

**Authors:** Sonia Shastri, Tanvi Shinde, Krystel L. Woolley, Jason A. Smith, Nuri Gueven, Rajaraman Eri

**Affiliations:** ^1^ Gut Health Laboratory, School of Health Sciences, College of Health and Medicine, University of Tasmania, Launceston, TAS, Australia; ^2^ Centre for Food Innovation, Tasmanian Institute of Agriculture, University of Tasmania, Launceston, TAS, Australia; ^3^ School of Natural Sciences-Chemistry, College of Science and Engineering, University of Tasmania, Hobart, TAS, Australia; ^4^ School of Pharmacy and Pharmacology, College of Health and Medicine, University of Tasmania, Hobart, TAS, Australia

**Keywords:** cytokines, endoplasmic reticulum stress, inflammatory bowel disease, naphthoquinones, tight junction proteins

## Abstract

Ulcerative colitis (UC) is characterised by chronic, relapsing, idiopathic, and multifactorial colon inflammation. Recent evidence suggests that mitochondrial dysfunction plays a critical role in the onset and recurrence of this disease. Previous reports highlighted the potential of short-chain quinones (SCQs) for the treatment of mitochondrial dysfunction due to their reversible redox characteristics. We hypothesised that a recently described potent mitoprotective SCQ (UTA77) could ameliorate UC symptoms and pathology. In a dextran sodium sulphate- (DSS-) induced acute colitis model in C57BL/6J mice, UTA77 substantially improved DSS-induced body weight loss, disease activity index (DAI), colon length, and histopathology. UTA77 administration also significantly increased the expression of tight junction (TJ) proteins occludin and zona-occludin 1 (ZO-1), which preserved intestinal barrier integrity. Similar responses were observed in the spontaneous Winnie model of chronic colitis, where UTA77 significantly improved DAI, colon length, and histopathology. Furthermore, UTA77 potently suppressed elevated levels of proinflammatory cytokines and chemokines in colonic explants of both DSS-treated and Winnie mice. These results strongly suggest that UTA77 or its derivatives could be a promising novel therapeutic approach for the treatment of human UC.

## Introduction

Ulcerative colitis (UC) and Crohn’s disease (CD) are the two basic forms of inflammatory bowel disease (IBD). Despite the unclear aetiology, environmental and genetic factors are known risk factors associated with the initiation of IBD (Khor et al., 2011). IBD patients usually present with clinical manifestations of bloody stool, diarrhoea, abdominal pain, fatigue, and body weight loss. Patients are also often at higher risk of developing extraintestinal diseases (spondylitis and cholangitis), colorectal cancer if left untreated, and other inflammatory diseases such as multiple sclerosis, bronchitis, renal diseases, and asthma (Bernstein et al., 2001; Bernstein et al., 2005; Lakatos et al., 2006). Current treatment approaches of IBD include the use of corticosteroids, biologicals, 5-aminosalicylates, and immunomodulators. However, none of these approaches are curative but are associated with a risk of adverse events such as steroid dependency, infertility, blood disorders, and liver diseases (Faubion et al., 2001; Carty and Rampton, 2003; Rutgeerts et al., 2005; Govani and Higgins, 2010; Stallmach et al., 2010; Oz et al., 2013). The limitations of current IBD therapies highlight the need to identify and test novel therapeutic strategies that aim to target causative pathophysiological pathways. Recently, advances in understanding the pathogenesis of UC support a pathophysiological circuit that involves reduced intestinal barrier integrity, dysregulated immune response, endoplasmic reticulum stress (ER stress), oxidative stress, and mitochondrial dysfunction, which cooperatively initiate and sustain the inflammation of the gut (Ott et al., 2004; Cobrin and Abreu, 2005; Targan and Karp, 2005; Gaya et al., 2006; Kaser and Blumberg, 2009; Tian et al., 2017; Holmberg et al., 2018). This new understanding opens the door to identify new therapeutic targets for the treatment of UC that interferes with one or several of the components of this vicious pathological cycle.

Several studies independently delineated the role of mitochondrial dysfunction in UC patients and in animal models of intestinal inflammation (Delpre et al., 1989; Söderholm et al., 2002; Nazli et al., 2004; Rodenburg et al., 2008; Dashdorj et al., 2013; Ho et al., 2018). Although the exact underlying mechanism for the mitochondrial dysfunction in the pathogenesis UC is unclear, it was suggested that abnormal mitochondrial morphology, oxidative stress, ER stress, and the presence of hypoxia play key roles. It was also established that maintenance of intestinal barrier integrity by tight junction (TJ) proteins is an energy-dependent process that requires mitochondrial adenosine triphosphate (ATP) (Roediger, 1980; Lewis and McKay, 2009). However, in UC, low levels of mitochondrial ATP synthesis in the intestinal mucosa (Kameyama et al., 1984; Schürmann et al., 1999) therefore can reduce the expression of TJ proteins, which disrupts barrier integrity and eventually leads to intestinal inflammation (Wang et al., 2014). Furthermore, mitochondria-derived oxidative stress in the intestinal epithelium significantly contributes to the progression of UC (Zhang et al., 2012; Wang et al., 2014; Tian et al., 2017). Mucosal inflammation decreases oxygen availability to intestinal epithelial cells to create a pathological state of hypoxia (Shah, 2016). Prolonged hypoxia further increases the production of mitochondrial reactive oxygen species (ROS) and proinflammatory cytokines in colitis (Shah, 2016). Excessive oxidative stress further destabilises mitochondria and damages DNA, protein, and lipids in the colonic plasma, which was demonstrated in tissues of colitic patients and preclinical mouse models of colitis (Du et al., 1998; Farhadi et al., 2005; Rezaie et al., 2007; Biasi et al., 2013; Dashdorj et al., 2013; Boyapati et al., 2018; Shastri et al., 2020a). Based on the tight functional and spatial interactions between mitochondria and endoplasmic reticulum (Giorgi et al., 2009), the presence of ER stress in goblet cells was confirmed in preclinical and clinical studies (Shkoda et al., 2007; Heazlewood et al., 2008; Kaser et al., 2008). In addition, ER stress was reported to play a significant role in the inflammatory process of UC (Heazlewood et al., 2008; Kaser et al., 2008; Das et al., 2013; Cao et al., 2016). A fragmented ER along with deteriorating mitochondria implicates both mitochondrial dysfunction and aberrant ER stress in the pathological cascade of UC (Cao et al., 2014). Many clinical and preclinical studies have described the altered secretion of cytokines by immune cells as a causative factor in the pathogenesis of UC (Powrie et al., 1994; Strober et al., 2002; Duerr et al., 2006; Alex et al., 2008; Franke et al., 2008; Strober and Fuss, 2011; Pearl et al., 2013; Nemeth et al., 2017), where mitochondrial function regulates the release proinflammatory cytokines such as interleukin beta and 18 (IL-1β and IL-18) through inflammasome activation (Dashdorj et al., 2013; Guo et al., 2015; Novak and Mollen, 2015). Therefore, simultaneous targeting of all pathophysiological factors by pharmacological targeting of mitochondrial function could be a promising new therapeutic approach for the treatment of UC.

Naphthoquinones, occurring either naturally or as synthetic derivatives, are gaining wider attention due to their mitoprotective, antioxidant, anticancer, and antiparasitic properties (Pinto and de Castro, 2009; Vos et al., 2012; Rahn et al., 2014; Pereyra et al., 2019). Natural naphthoquinones have been reported to possess potent anti-inflammatory properties (Andújar et al., 2012; Fan et al., 2013; Pile et al., 2013; Lomba et al., 2017; Sun et al., 2019). As such, 1,4-naphthoquinones inhibited tumor necrosis factor-alpha (TNF-α) in lipopolysaccharide- (LPS-) induced acute inflammation in mice and proved to be more potent than benzoquinones (Kobayashi et al., 2011). The derivatives of 1,4-naphthoquinones also reduced the expression of IL-1β, interleukin 6 (IL-6), and TNF-α in a murine macrophage cell line (Liu et al., 2020). The naphthoquinone shikonin and its derivatives attenuated inflammation by reducing IL-6, TNF-α, and IL-1β levels in the colon and serum of colitic mice (Andújar et al., 2012). Furthermore, shikonin also protected TJ proteins occludin and claudin-1 and preserved the intestinal barrier integrity in DSS-induced colitis (Andújar et al., 2012). In addition to its anti-inflammatory property, shikonin also suppressed ER stress in isoproterenol-induced heart injury (Yang et al., 2017), while another naphthoquinone, plumbagin, inhibited intestinal inflammation by downregulating proinflammatory cytokines, which was associated with improved disease histology (Pile et al., 2013). Recently, more than 148 novel naphthoquinones derivatives of short-chain quinones (SCQs) have been synthesised and screened *in vitro* for their cytoprotective activity against mitochondrial dysfunction (Woolley et al., 2019). Among them, 16 amide-linked naphthoquinones showed high metabolic stability, low toxicity, and better cytoprotection *in vitro* (Woolley et al., 2019; Feng et al., 2020a; Feng et al., 2020b). Indeed, one of the newly synthesised naphthoquinones (UTA77) restored vision loss *in vivo* in mitochondrial dysfunction-related pathology, i.e., diabetic retinopathy (Daniel et al., 2021).

**FIGURE 1:**
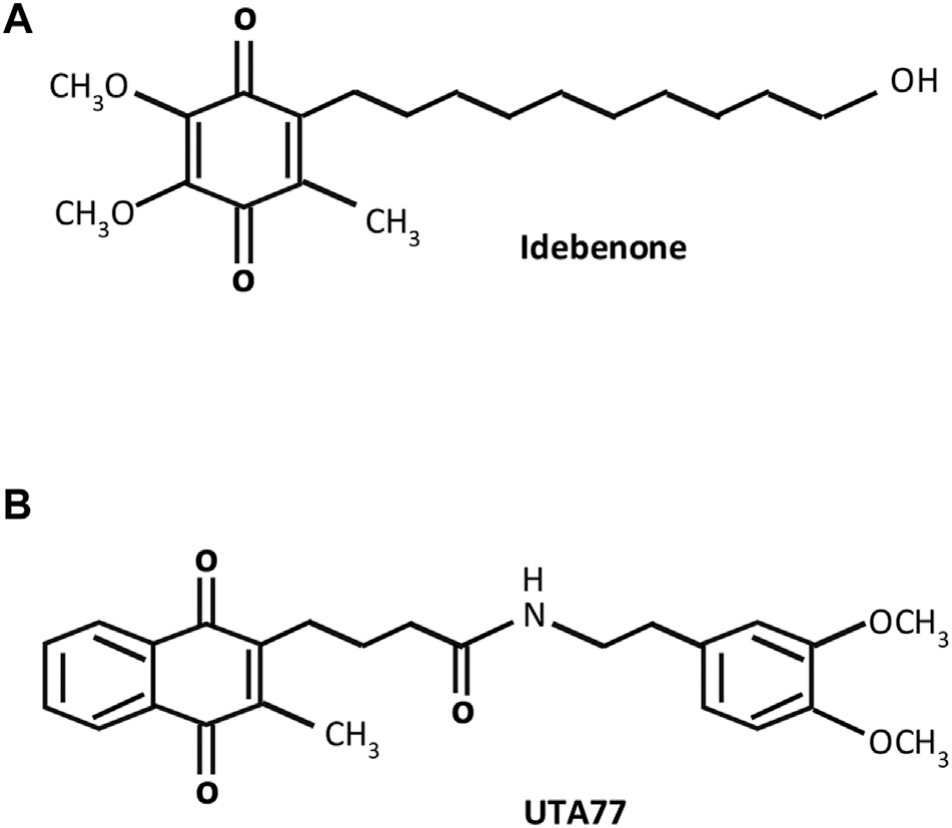
Molecular structures of short-chain quinones (SCQs). **(A)** Benzoquinone idebenone and **(B)** novel naphthoquinone UTA77.

We previously showed that a benzoquinone-based SCQ (idebenone) (Figure 1A) ameliorated intestinal inflammation by exerting antioxidant and anti-inflammatory effects in models of acute and chronic colitis (Shastri et al., 2020a; Shastri et al., 2020b). Therefore, this study hypothesised that a newly developed mitoprotective naphthoquinone-based SCQ (UTA77) (Figure 1B) could be an effective therapeutic drug candidate to combat intestinal inflammation by simultaneously targeting multiple pathophysiological factors.

For the first time, this study utilised two validated mouse models of UC (DSS-induced acute colitis model and Winnie mouse model of spontaneous chronic colitis), to assess the therapeutic potential of a novel mitoprotective naphthoquinone (UTA77). The results of the current study consistently demonstrate significant protection by UTA77 in both models. UTA77 reduced intestinal inflammation by directly suppressing proinflammatory cytokines and by preserving intestinal barrier integrity.

## Materials and Methodology

### Animals

Ten female C57BL/6J and six Winnie mice of both sexes were obtained from animal breeding facility of the University of Tasmania. All the animals were housed in a controlled temperature environment with 12 h day and night cycle. Before including in any experiment, all the animals had undergone through acclimatisation period of 7 days. During acclimatisation, all the mice were caged individually with proper access to autoclaved drinking water *ad libitium* and normal chow pellets. Also, individual body weights were recorded daily during the acclimatisation period. All the animal trials were performed in accordance with the ethics approval from the Animal Ethics Committee of University of Tasmania (approval number: A00016166 approved on March 6, 2017). All the procedures were conducted in accordance with the Australian Code of Practise for Care and Use of Animals for Scientific Purposes (eighth Edition, 2013).

### Study Experimental Design and Drug Treatment

After 1 week of acclimatisation, all the C57BL/6J (aged 7 weeks) were randomly allocated into three groups: 1) healthy controls without DSS and treatment (HC), 2) DSS controls without treatment (DSS), and 3) UTA77-treated group (DSS + UTA77) with 10 animals/group. In case of chronic colitis Winnie model, groups were divided as follows: healthy controls (C57BL/6J) without treatment (HC), 2) Winnie controls without treatment (Win), and 3) UTA77-treated Winnie (Win + UTA77) having six animals/group. UTA77 (provided by UTAS) was administered orally at a dose of 200 mg/kg of bodyweight of mice. For drug preparation, UTA77 was suspended in 0.5% of carboxymethyl cellulose (CMC) and 4% sucrose and mixed with autoclaved powdered chow pellets. The wet food mash of UTA77 was aliquoted as 2.5 g/dish and stored in −20°C. For DSS-induced acute colitis, 2.5% of DSS with a molecular weight of 30,000–50,000 KDa (MP Biomedicals, NSW, Australia) was administered continuously for 7 days in autoclaved drinking water to all the C57BL/6J groups except HC. The vehicle and UTA77 were also coadministered orally for 7 days continuously to DSS controls and DSS + UTA77-treated groups, respectively. In case of spontaneous chronic colitis, UTA77 was administered to UTA77-treated Winnie and vehicle to Winnie controls for continuously 21 days provided with autoclaved normal drinking water to all groups.

### Histopathological Analysis and Clinical Scoring

Disease Activity Index (DAI) was calculated daily by adding the individual scores of body weight, bloody stool, and stool consistency over the whole period of the experiment. Briefly, scoring was done as described previously (Shinde et al., 2019; Shastri et al., 2020a): body weight loss (score 0 = %, score 1 = 1–5%, score 2 = 6–10%, and score 3 = 11–15%), stool consistency (score 0 = hard/formed stool, score 1 = soft/loose stool, score 2 = very soft, and score 3 = watery stool/diarrhoea), and bloody stool (score 0 = negative haemoccult/no traces of blood, score 1 = positive haemoccult, score 2 = visible traces of blood, and score 3 = gross/rectal bleeding). The colons were taken out, opened, and bisected longitudinally into two halves on day 8 (acute colitis model) and day 21 (spontaneous chronic colitis model) after dissection. One half was stored for histopathological analysis using the Swiss roll technique, while the remaining another half was collected and snap-frozen for further molecular analysis. Swiss rolls were fixed in 10% v/v neutral-buffered formalin for 24 h and later transferred to 70% ethanol before being embedded in paraffin wax. 5 μm tissue sections embedded in paraffin wax were cut using microtome. Paraffin-embedded tissue slides were then stained with haematoxylin and eosin (H&E) dyes and were used for evaluating histopathological grading in a blinded manner as described previously for DSS-induced acute colitis and spontaneous chronic colitis in Winnie (Perera et al., 2018). Images were captured using Leica microscope (DM500, Leica Microsystems, Mannheim, Germany).

### Alcian Blue Staining

Alcian Blue staining was performed to visualise the sulphated and acidic mucopolysaccharide mucin (MUC2) secretion from goblet cells using Alcian Blue staining kit pH 2.5 (ab150662, Abcam, Australia) as described previously (Sovran et al., 2016; Shinde et al., 2019; Shastri et al., 2020a). Briefly, tissue embedded slides were dewaxed in xylene and rehydrated in series of graded ethanol in descending order (100, 100, 95, and 70%). Slides were first incubated with Alcian Blue stain for 30 min at room temperature before counterstained with Safranin O for 5 min. Stained slides were further dehydrated in graded ethanol in ascending order (70, 95, 100, and 100%) and cleared in xylene before mounting with DPX medium (Sigma-Aldrich, NSW, Australia). Image Pro-Plus seven software (Media Cybernetics, Inc., Rockville, Maryland, United States) was used to analyse the staining intensity by randomly capturing images (DM500, Leica Microsystems, Mannheim, Germany) from four different area/slides (*n* = 3 slides/group) in a blinded fashion.

### Immunohistochemistry

Immunohistochemical analysis was performed to detect the localisation of TJ proteins occludin and ZO-1 using an HRP/DAB Detection IHC kit (ab64621, Abcam, Australia) as mentioned previously (Shastri et al., 2020a; Shastri et al., 2020b) and according to manufacturer’s instructions. Briefly, paraffin-embedded tissue section slides were dewaxed in xylene and rehydrated through series of graded ethanol before incubating in citrate buffer (pH-6) for antigen retrieval process in a decloaking chamber at 121°C for 4 min. The endogenous peroxidase activity was blocked by incubating the tissue slides with hydrogen peroxide block for 10 min, following protein blocking for further 30 min at room temperature. Primary antibodies against occludin (1:600, NBP-1–87,402, Novus Biologicals, Victoria, Australia) and ZO-1 (1:400, NBP1-85046, Novus Biologicals, Victoria, Australia) were used to incubate colonic tissue slides overnight at 4°C. After washing the slides in phosphate buffer saline (1X PBS), subsequently, slides were first incubated with biotinylated goat anti-rabbit IgG for 10 min, followed by incubation with Streptavidin-peroxidase conjugate for further 10 min at room temperature. Slides were further incubated using DAB chromogen and substrate for 10 min according to the manufacturer’s protocol. Slides were counterstained with haematoxylin and bluing in ammonia water before dehydrating in graded ethanol and clearing in xylene. Finally, the slides were mounted using DPX media, and images were taken using Leica DM500 microscope. Image Pro-Plus 7.0 software was used to examine the staining intensity by randomly choosing four different areas from one slide where (*n* = 3 slides/group), in a blinded manner.

### Lipid Peroxidation

The amount of malondialdehyde (MDA) levels present in colonic tissue was determined using lipid peroxidation colorimetric/fluorometric assay kit (K739, Bio Vision, NSW, Australia) as described previously (Das et al., 2014). The colon tissues were homogenised in lysis buffer provided in the kit followed by centrifugation at 13,000 x g to collect the supernatant. Afterwards, thiobarbituric acid (TBA) was added to the resulting supernatant which formed MDA-TBA adduct after boiling at 95°C for 1 h. The absorbance of coloured MDA-TBA adduct was detected colorimetrically at a wavelength of 532 nm in a spectrophotometer. The amount of MDA in the tissue samples was quantified by plotting a graph against MDA standard calibration curve provided in the kit. The values were expressed as nmol/mg protein.

### Superoxide Dismutase Activity

The percentage of total superoxide dismutase activity in colonic tissues was calculated using a commercially available superoxide dismutase activity assay kit (ab65354, Abcam, Australia) as mentioned earlier (Shastri et al., 2020a). Briefly, followed by homogenisation of colonic tissues in ice-cold Tris/HCl (pH -7.4, 0.1 M containing Triton X-100, phenylmethylsulfonyl (PMSF) and β-mercaptoethanol) and centrifugation for 5 min at 14,000 x g, supernatants containing total SOD activity of cytosolic as well as mitochondrial SOD were collected. The detection depends upon the formation of yellow formazan crystals upon reduction with superoxide anions by adding WST-1 (water-soluble tetrazolium-1) to the collected supernatant. The data were calculated by measuring the absorbance at 450 nm colorimetrically where SOD activity represents the percentage inhibition of superoxide production. The more the SOD in the sample, the more the inhibition activity.

### Nitric Oxide Production

Griess reagent kit (G2930, Promega, Victoria, Australia) was used to detect the nitrite (by-product of nitric oxide (NO)) levels as described previously (Shastri et al., 2020a). The assay was performed according to the manufacturer’s instructions. The nitrite standards at various concentrations (100, 50, 225, 12.5, 6.25, 3.13, 1.56, and 0 μM) and colonic tissue explants were pipetted into a 96-well plate and incubated according to instructions. The absorbance of the sample was plotted against nitrite reference curve at 550 nm. The values were expressed as a concentration in μM/Gram of tissue.

### Western Blotting Analysis

Briefly, frozen colon tissue sections were lysed in RIPA buffer containing protease inhibitor cocktails (Complete ULTRA Tablets, Mini, EDTA-free, NSW, Australia). The concentration of protein in supernatants was determined according to the protocol described by DC protein assay kit from Bio-Rad. Tissue-extracted proteins lysates (20 μg) were suspended in loading buffer and separated on SDS-PAGE polyacrylamide gels (4–20%, Mini-Protean TGX Precast Gels, Bio-Rad, NSW, Australia) in 1x electrophoresis buffer run at 100 V for 1 h. Subsequently, proteins from gel were electro-transferred onto polyvinylidene difluoride membrane (PVDF) using a wet transfer system. The membranes were then blocked in 5% skim milk prepared in TBST and incubated overnight with primary antibody against GRP78 (1:1,000, NBP1-06274, Novus biologicals), CHOP (1:1,000, NBP2-13172, Novus Biologicals), and β-actin (1:8,000, NB600-503, Novus Biologicals) at 4°C. The antibodies were detected by incubating with Horseradish peroxidase- (HRP-) conjugated secondary antibody (1:3,000, 7,074, Cell Signaling Technology, Australia) at room temperature for 1 h. Protein bands were visualised using SuperSignal, West Pico PLUS chemiluminescent substrate (Thermo Scientific, Victoria, Australia). The images were captured and band density relative to housekeeping gene (β-actin) was measured using LAS-3000 image reader version 2.2 (Fujifilm Luminescent Image Analyzer, Fuji Life Sciences, Japan).

### Cytokine Measurement

Explants from proximal and distal colon tissue from each group were excised, washed with PBS, and cultured in RPMI 1640 culture medium containing 1% antibiotics solution (10 mg/ml streptomycin and 10,000 U/ml penicillin, Sigma-Aldrich Pty. Ltd. NSW, Australia) and 10% v/v fetal bovine serum (Gibco Life Technologies Pty. Ltd. Melbourne, Australia) in a 12-well plate as described previously (Lean et al., 2015; Shinde et al., 2019). After overnight incubation, the supernatant was collected, centrifuged, and stored at −80°C until further analysis. Cytokine concentrations in the colonic explants were determined using Bio-Plex Pro Mouse cytokine 23-plex kit (catalogue number: #M60009RDPD, Bio-Rad, NSW, Australia) according to manufacturer’s protocol using an instrument Bio-Plex 200 (Bio-Rad Laboratories). Bio-Plex Manager software version six was used to analyse the cytokine concentrations and presented as pg/mL/g of tissue. The cytokines values were normalised to tissue weight by dividing observed cytokine concentration (pg/ml) with tissue weight in grams.

### Statistical Analysis

Statistical analysis was performed using GraphPad Prism Software version 8.0, CA, United States). All the data were presented as average mean value ± standard error of mean (SEM) of independent experiments carried out in triplicate. One-way analysis of variance (ANOVA) followed by Tukey’s post hoc test was used to evaluate the statistical difference between the three groups. For comparison between the two groups, nonparametric, unpaired, two-tailed *t*-test was performed. For analysis of body weight change and DAI, two-way ANOVA followed by Tukey’s post hoc test was used. Data were considered significant when *p* < 0.0001 (****), *p* < 0.001 (***), *p* < 0.01 (**), and *p* < 0.05 (*).

## Results

### Treatment With UTA77 Reduces Clinical Symptoms of Dextran Sodium Sulphate-Induced Acute Colitis

**FIGURE 2:**
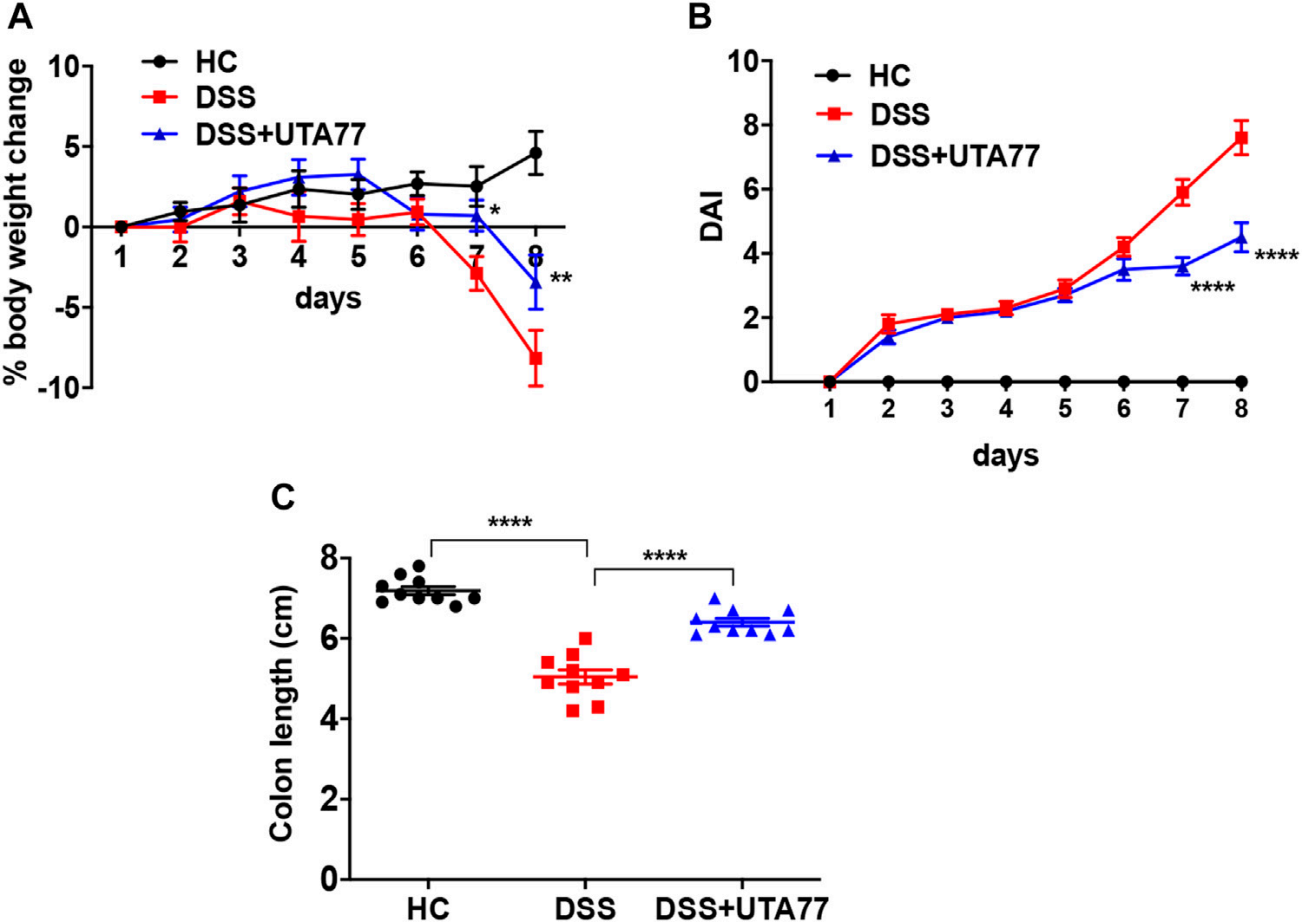
Effect of UTA77 on the pathology of DSS-induced experimental colitis. **(A)** % body weight change; **(B)** disease Activity Index (DAI). Statistical significance among groups evaluated by two-way ANOVA followed by Tukey’s posttest. *p < 0.05, **p < 0.01, and ****p < 0.0001 vs. DSS. **(C)** Colon length evaluated by one-way ANOVA followed by Tukey’s posttest. Data expressed as mean ± SEM (*n* = 10/group).

Induction of DSS for 7 days in mice resulted in a drastic increase in colonic inflammation, evident by severe body weight loss (−8.1 ± 1.7%) with higher DAI (7.6 ± 0.5) at the end of the experiment on day 8, while healthy mice in the control (HC) group maintained a stable body weight and DAI during the observation period (Figures 2A,B). In contrast, UTA77 intervention protected against body weight loss from day 7 (*p < 0.05*) till day 8 (−3.4 ± 1.7%, *p < 0.01*) in comparison to the untreated DSS-control group. UTA77 treatment also significantly (*p < 0.001*) reduced the DAI (which is the integrated index of bloody stool/stool consistency and body weight loss) on days 7 and 8 (4.5 ± 0.5), when compared to the untreated DSS-control group (Figure 2B). Furthermore, macroscopic evaluation of colon segments on day 8 unveiled a significant colon shortening by DSS administration (5.0 ± 0.2 cm) in comparison to the HC group (7.2 ± 0.1 cm). Consistent with the previous results, UTA77 administration also significantly protected against colon shortening (6.4 ± 0.1 cm) (Figure 2C).

### Treatment With UTA77 Prevents Histopathology of Acute Colitis

**FIGURE 3:**
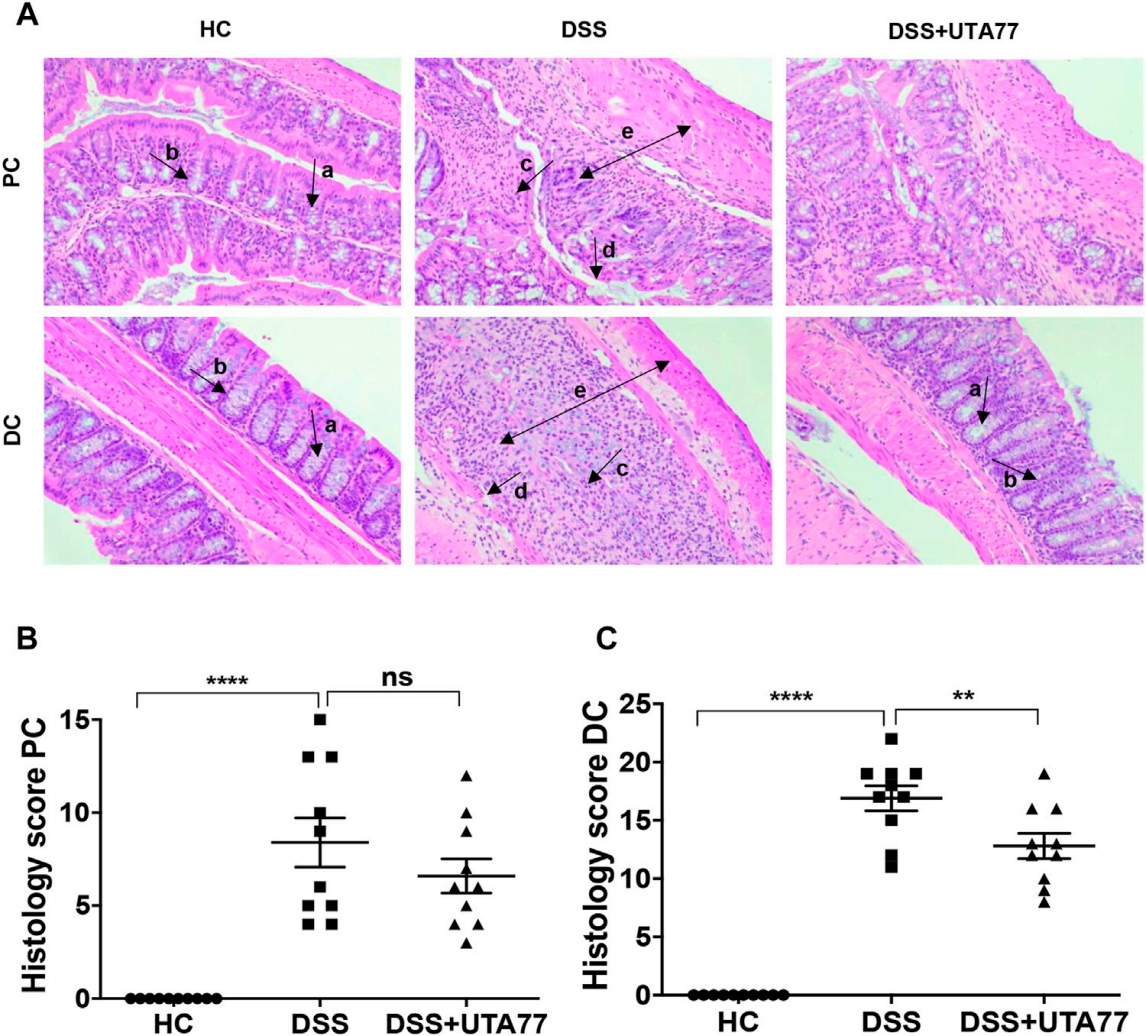
Effect of UTA77 on histopathology in DSS-induced colitis. **(A)** Histological representation of proximal colon (PC) and distal colon (DC) sections stained with H&E for healthy mice (HC), DSS-control mice (DSS), and DSS + UTA77-treated mice (DSS + UTA77) at ×20 magnification. **(B)** and **(C)** represent the histopathology scores for each animal calculated after microscopic analysis of tissue sections from the PC and DC. Statistical significance among groups was evaluated by one-way ANOVA followed by Tukey’s posttest. **p < 0.01, ****p < 0.0001, and ns: nonsignificant. Data expressed as mean ± SEM (*n* = 10/group). Arrows indicate goblet cells (a), crypts/regeneration of crypts (b), inflammatory cells infiltration (c), epithelium surface erosion (d), and submucosal oedema (e).

The H&E staining of mouse proximal colon (PC) and distal colon (DC) sections showed extensive histological damage upon DSS induction (Figure 3A). Colon tissues of the untreated DSS-control group showed a severe loss of crypts, epithelial erosion, submucosal oedema, infiltration of inflammatory cells, and goblet cells loss, which were more prominent in DC than PC. While the cumulative histological score for the untreated DSS-control group was 16.9 ± 0.8 for the DC (Figure 3C) and 8.4 ± 1.3 for the PC (Figure 3B), histological scores for the DC and PC were 0 for the HC group, as they showed no sign of colonic damage. UTA77 treatment substantially protected against damage of colonic crypts and goblet cells, infiltration of inflammatory cells, and loss of epithelial structure. This resulted in a significant reduction (*p < 0.01*) of the cumulative histological score (12.8 ± 1.1) for the DC in comparison to the untreated DSS-control group. In contrast, the UTA77 treatment showed no significant protection in the PC as demonstrated by a histological score of 6.6 ± 0.9.

**FIGURE 4:**
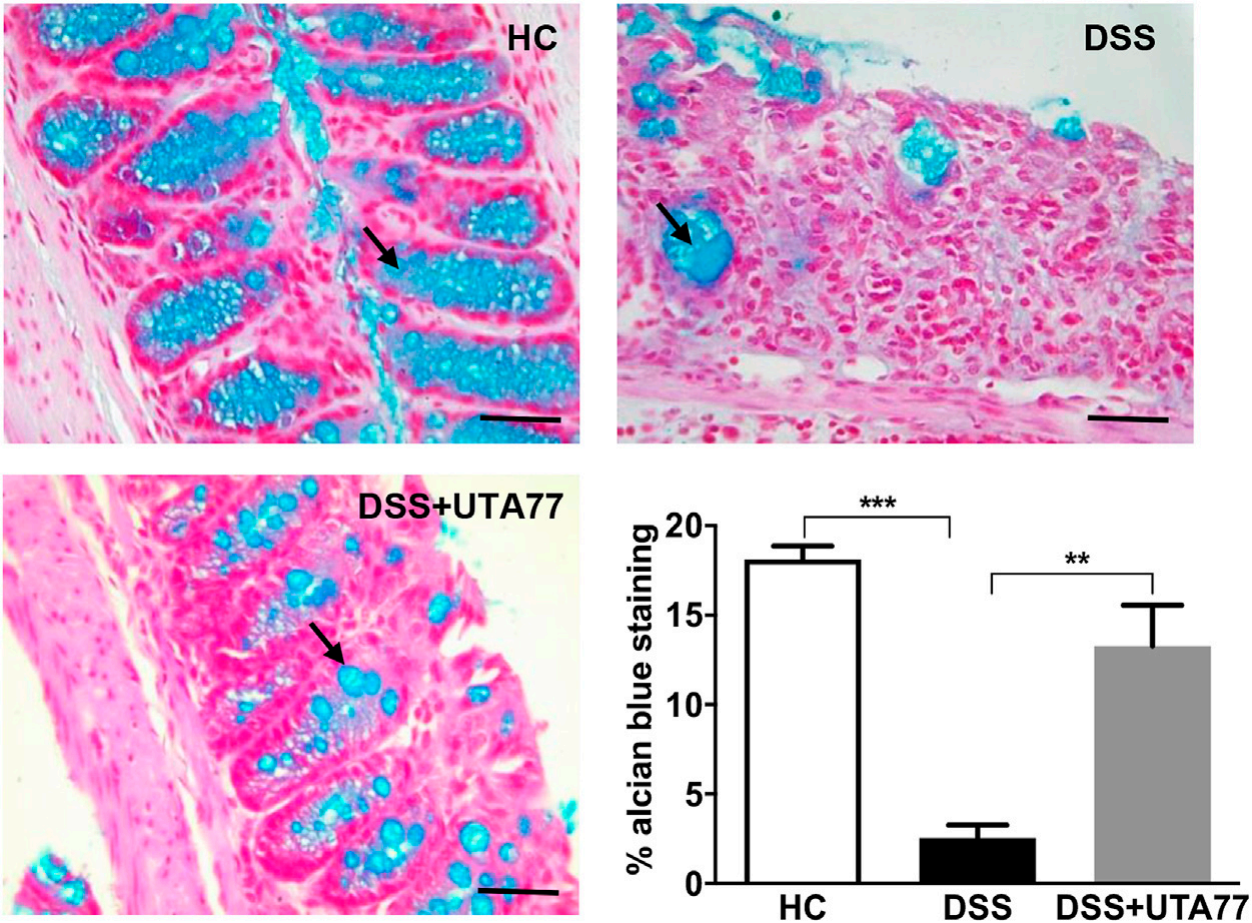
Effect of UTA77 on goblet cells. Goblet cells producing mucus stained with Alcian Blue dye for HC, DSS, and DSS + UTA77 groups in distal colon along with graphical representation of staining intensity of Alcian Blue dye for each group *(n* = 3/group). Statistical significance among groups evaluated by one-way ANOVA followed by Tukey’s posttest where **p < 0.01 and ***p < 0.001. Images at ×40 magnification.

To examine the protective effect of UTA77 on goblet cells, Alcian Blue staining was performed. A substantial increase in mucus staining (blue) in the goblet cells was observed in the UTA77-treated DSS group, whereas in the untreated DSS-control group where goblet cells were lost, staining was almost negligible (Figure 4).

### UTA77 Maintains Intestinal Barrier Integrity by Preserving the Expression of TJ Proteins

**FIGURE 5:**
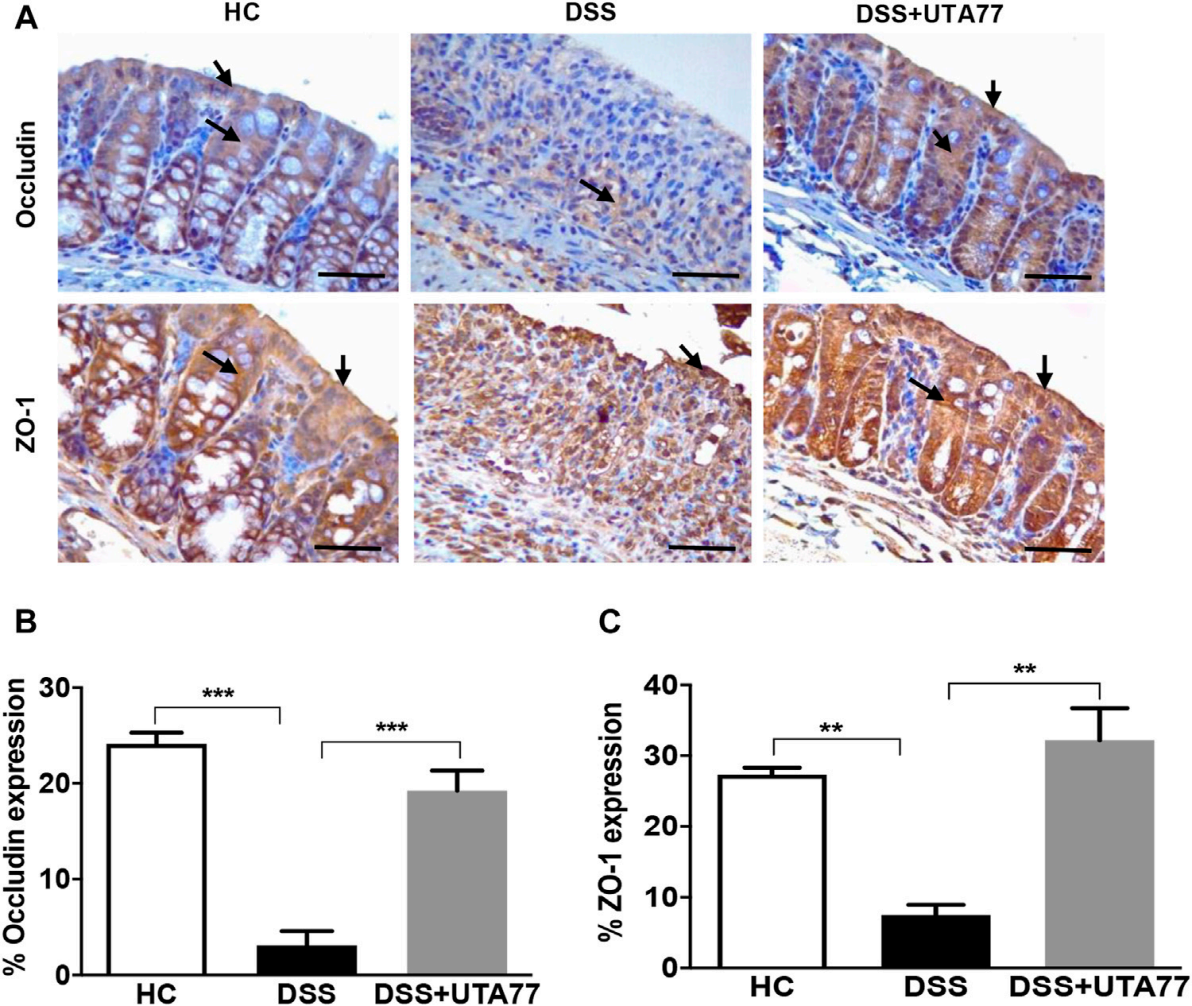
Effect of UTA77 on tight junction (TJ) protein expression in DSS-induced experimental colitis. **(A)** Immunohistochemical analysis of occludin and ZO-1, **(B)** average occludin expression in distal colon, and **(C)** average ZO-1 expression in distal colon. Data expressed as mean ± SEM (*n* = 3/group) and statistical significance evaluated by one-way ANOVA followed by Tukey’s posttest where **p < 0.01 and ***p < 0.001. Images at ×40 magnification. Arrow indicates localisation of staining.

To investigate the protective effect of UTA77 on intestinal barrier integrity, the expression of the TJ proteins occludin and ZO-1 was quantified on the colon section. In the HC group, staining for both TJ proteins occludin and ZO-1was intense and localised at both apical and basolateral surface of the crypts, while in the untreated DSS-control group, occludin and ZO-1 staining were very weak (Figures 5A,B). In contrast, the UTA77 treatment effectively restored the expression of TJ proteins to a level comparable to the HC group.

### UTA77 Attenuates pro-Inflammatory Cytokine Levels in Acute Colitis

**FIGURE 6:**
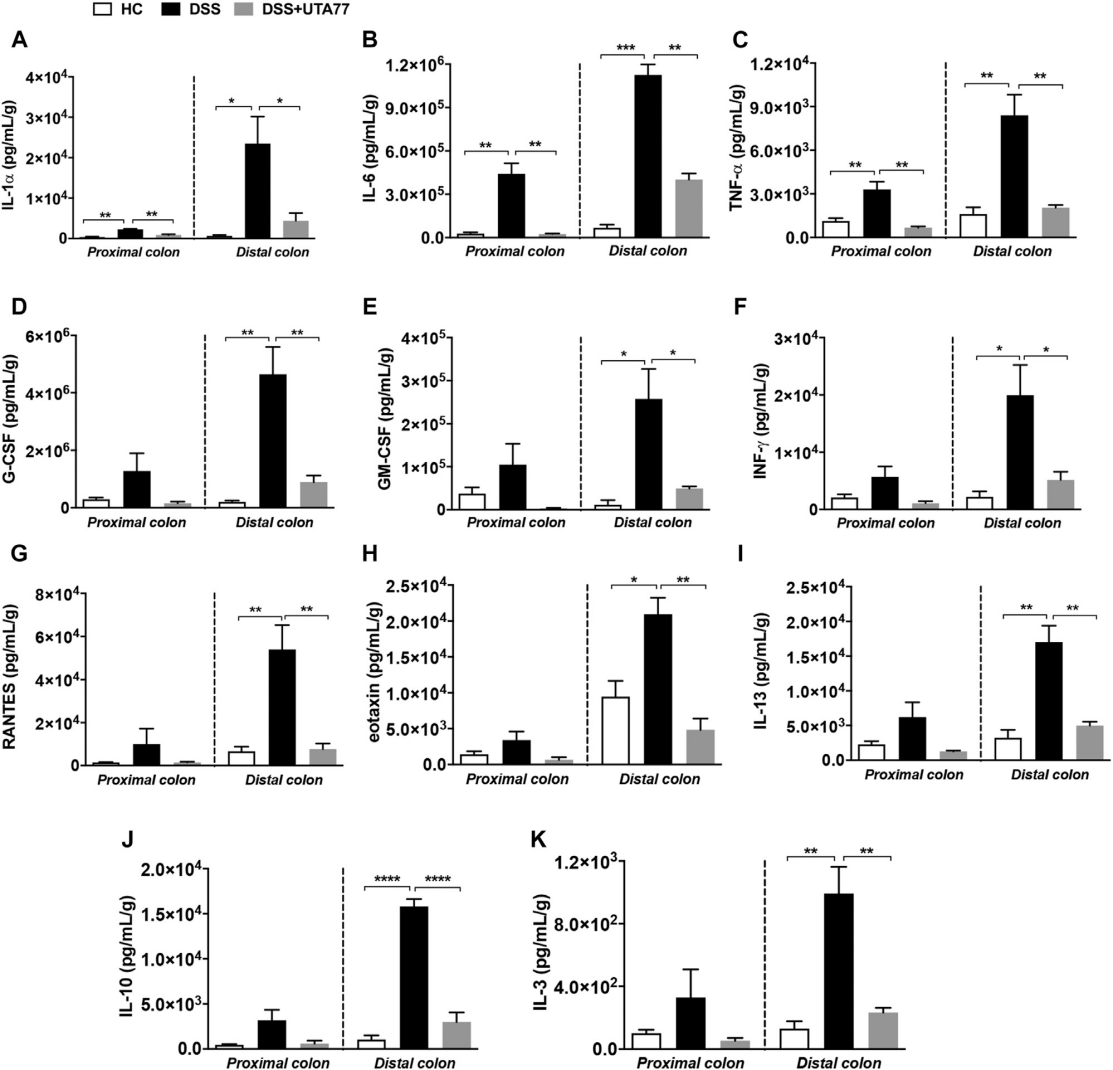
Effect of UTA77 on the level of inflammatory cytokines and chemokines in colon tissue. Tissue levels of **(A)** IL-1α, **(B)** IL-6, **(C)** TNF-α, **(D)** G-CSF, **(E)** GM-CSF, **(F)** INF-γ, **(G)** RANTES, **(H)** eotaxin, **(I)** IL-13, **(J)** IL-10, and **(K)** IL-3 in PC and DC were quantified by Bio-Plex assay. Data expressed as mean ± SEM (*n* = 3/group) and statistical significance evaluated by one-way ANOVA followed by Tukey’s posttest where *p < 0.05, **p < 0.01, ***p < 0.001, and ****p < 0.0001.

It is well established that dysregulation of intestinal cytokines is implicated during the pathogenesis of intestinal inflammation. Therefore, this study also explored the anti-inflammatory potential of UTA77 by assessing a range of cytokines. DSS-treatment increased the levels of several proinflammatory cytokines in the PC and DC compared to the HC group (Figure 6). Compared to the untreated DSS-control group, UTA77 administration reduced the elevated levels of proinflammatory cytokines IL-1α, IL-6, TNF-α, G-CSF (granulocyte colony-stimulating factor), GM-CSF (granulocyte-macrophage colony-stimulating factor), INF-γ (interferon-gamma), RANTES (Regulated on Activation, Normal T Expressed and Secreted), eotaxin, IL-13, IL-3, and anti-inflammatory cytokine IL-10 to the levels of the HC group. However, no significant reduction of G-CSF, MIP-1β (macrophage inflammatory protein one beta), and RANTES in PC and INF-γ in DC was detected by administration of UTA77. No significant effects by UTA77 on several other cytokines were observed (Supplementary Figure S1).

### UTA77 Attenuates Colonic Inflammation in Spontaneous Chronic Colitis

**FIGURE 7:**
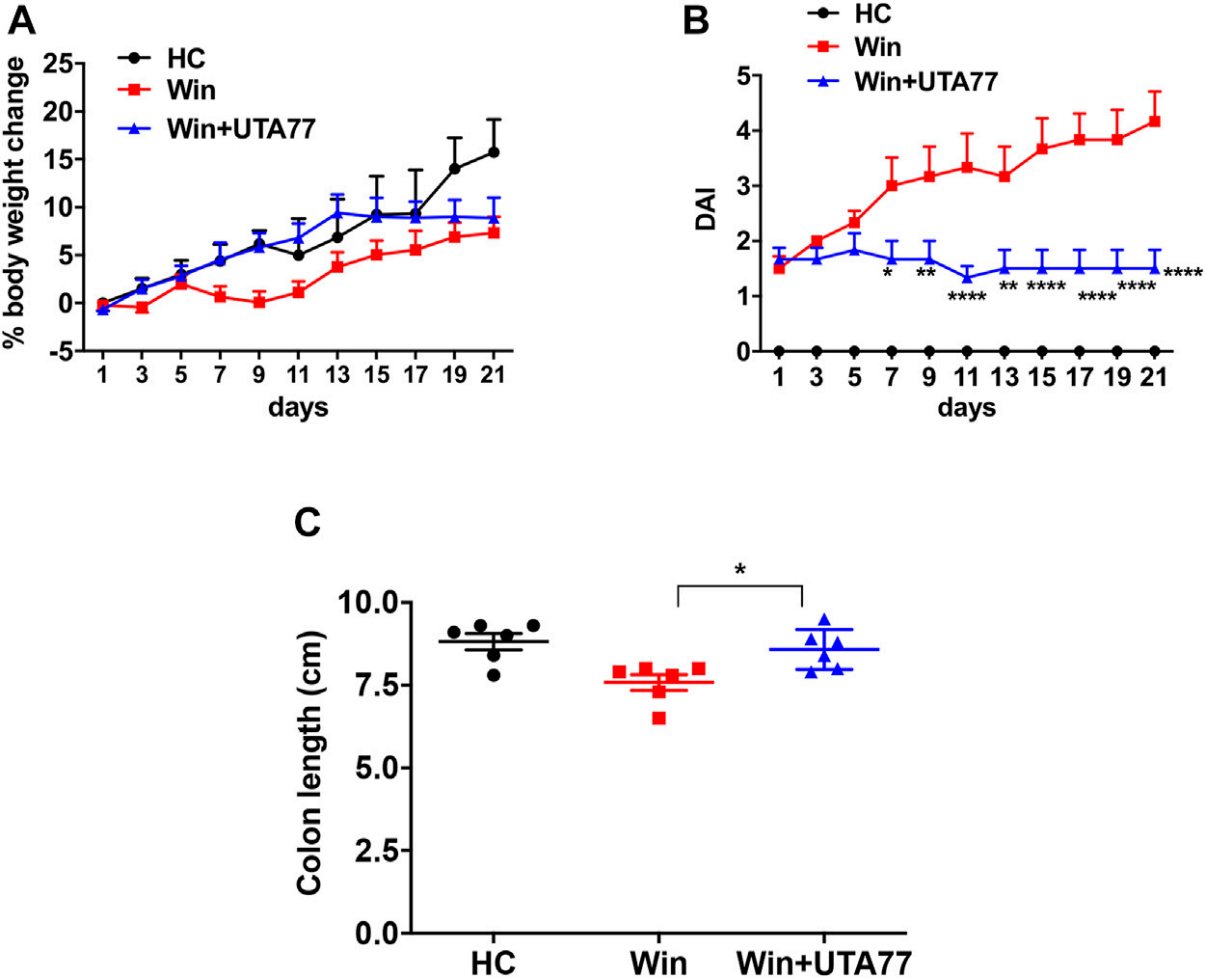
Effect of UTA77 on the pathology of spontaneous chronic colitis in Winnie mice. **(A)** % body weight change and **(B)** DAI of healthy controls (HC), Winnie controls (Win), and UTA77-treated Winnie group (Win + UTA77). Statistical significance among groups evaluated by two-way ANOVA followed by Tukey’s posttest. *p < 0.05, **p < 0.01, ***p < 0.001, and ****p < 0.0001 vs. Winnie*.* **(C)** Colon length evaluated by one-way ANOVA followed by Tukey’s posttest. Data expressed as mean ± SEM (*n* = 6/group).

**FIGURE 8:**
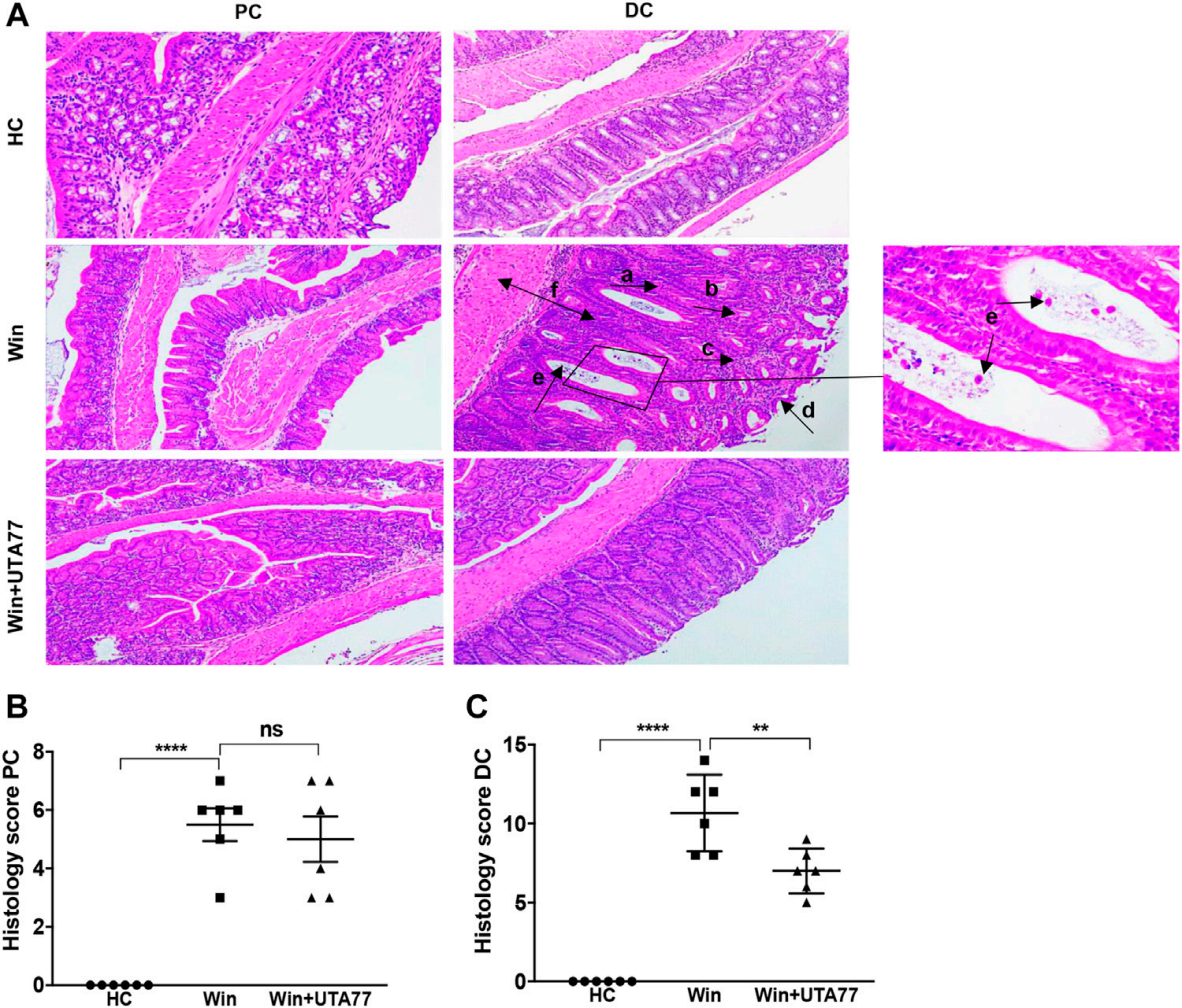
Effect of UTA77 on histopathology in spontaneous chronic colitis in Winnie mice. **(A)** Histological representation of PC and distal colon (DC) sections stained with H&E for healthy mice (HC), Winnie control mice (Winnie), and Winnie plus UTA77-treated mice (Winnie + UTA77) at ×10 magnification. **(B)** and **(C)** represent the histopathology scores for each animal calculated after microscopic analysis of tissue sections from the PC and DC. Statistical significance among groups was evaluated by one-way ANOVA followed by Tukey’s posttest. **p < 0.01, ****p < 0.0001, and ns: nonsignificant. Data expressed as mean ± SEM (*n* = 6/group). Arrows indicate goblet cells loss (a), crypts loss/crypts distortion (b), inflammatory cells infiltration (c), epithelial surface erosion (d), crypt abscesses (e), and mucosal/submucosal oedema (f).

Since the DSS-induced acute colitis model showed remarkable protection against colitis by UTA77, we also examined the effects of UTA77 in the Winnie mouse model of spontaneous chronic colitis. Compared to untreated Winnie control mice (Win), which had gained 7.3% body weight on day 21, UTA77-treated Winnie (Win + UTA77) gained on average 8.9% of body weight, which was not significantly different (Figure 7A). However, UTA77 treatment significantly improved the DAI score from day 7 (*p < 0.05*) with the highest significance level from day 15 onwards (*p < 0.0001*) (Figure 7B). At the end of the observation period, the colon of untreated Winnie mice was visibly inflamed, thickened, and shortened compared to healthy animals (HC). Consistent with the results from the acute colitis model, UTA77 significantly (*p < 0.05*) increased colon length in the UTA77-treated Winnie mice (8.6 ± 0.2 cm) compared to the Winnie control group (7.6 ± 0.2 cm) (Figure 7C). Histological analysis revealed a distorted crypt architecture, crypt abscesses with neutrophils inside the lumen, mucosal surface erosion, goblet cell loss, and infiltration of inflammatory cells in the lamina propria mainly in the DC of untreated Winnie mice (Figure 8A). These histological results were reflected in a high histological score of 10.7 ± 0.9 for the DC in untreated Winnie mice (Figure 8C), whereas UTA77-treated Winnie mice showed a significantly (*p < 0.01*) lower histological score of 7.0 ± 0.5. Comparatively, the histological score for UTA77-treated Winnie mice in the PC was low (5.0 ± 0.8) and not significantly different from untreated Winnie mice (5.5 ± 0.6) (Figure 8B).

### UTA77 Reduces a Marker of Intestinal ER Stress

**FIGURE 9:**
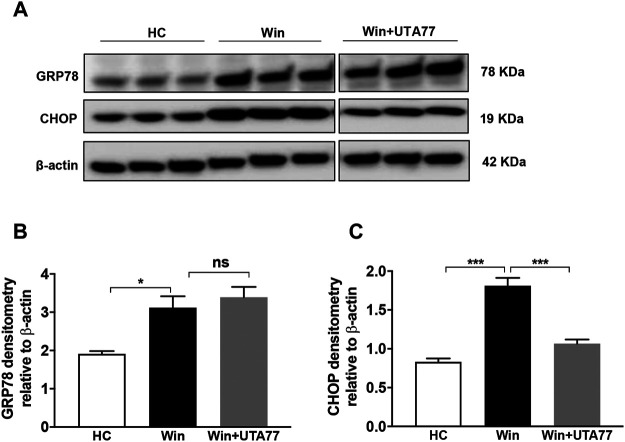
Effect of UTA77 on endoplasmic reticulum stress (ER stress) markers in spontaneous chronic colitis in Winnie mice. **(A)** Protein levels of GRP78 and CHOP were analysed using Western blotting in distal colon. **(B, C)** Densitometry of GRP78 and CHOP. Bands densities were normalised to the levels of β-actin. Data expressed as mean ± SEM (*n* = 3/group) using one-way ANOVA followed by Tukey’s posttest, where *p < 0.05, ***p < 0.001, and ns: nonsignificant. The controls used (HC and Win) in this figure have been published previously (Shastri et al., 2020b).

The Winnie model is associated with pronounced ER stress, which is responsible for the observed pathology. Therefore, the expression of ER stress markers glucose-regulated protein 78 (GRP78) and C/EBP homologous protein (CHOP) was assessed (Figure 9A). In untreated Winnie mice, GRP78 (*p < 0.05*) and CHOP (*p < 0.001*) proteins were significantly upregulated, compared to the HC group. UTA77 treatment significantly downregulated CHOP protein expression in Winnie (*p < 0.001*) but did not affect GRP78 expression (Figures 9B,C).

### UTA77 Prevents Elevated Levels of Pro-Inflammatory Cytokines in Chronic Colitis

**FIGURE 10:**
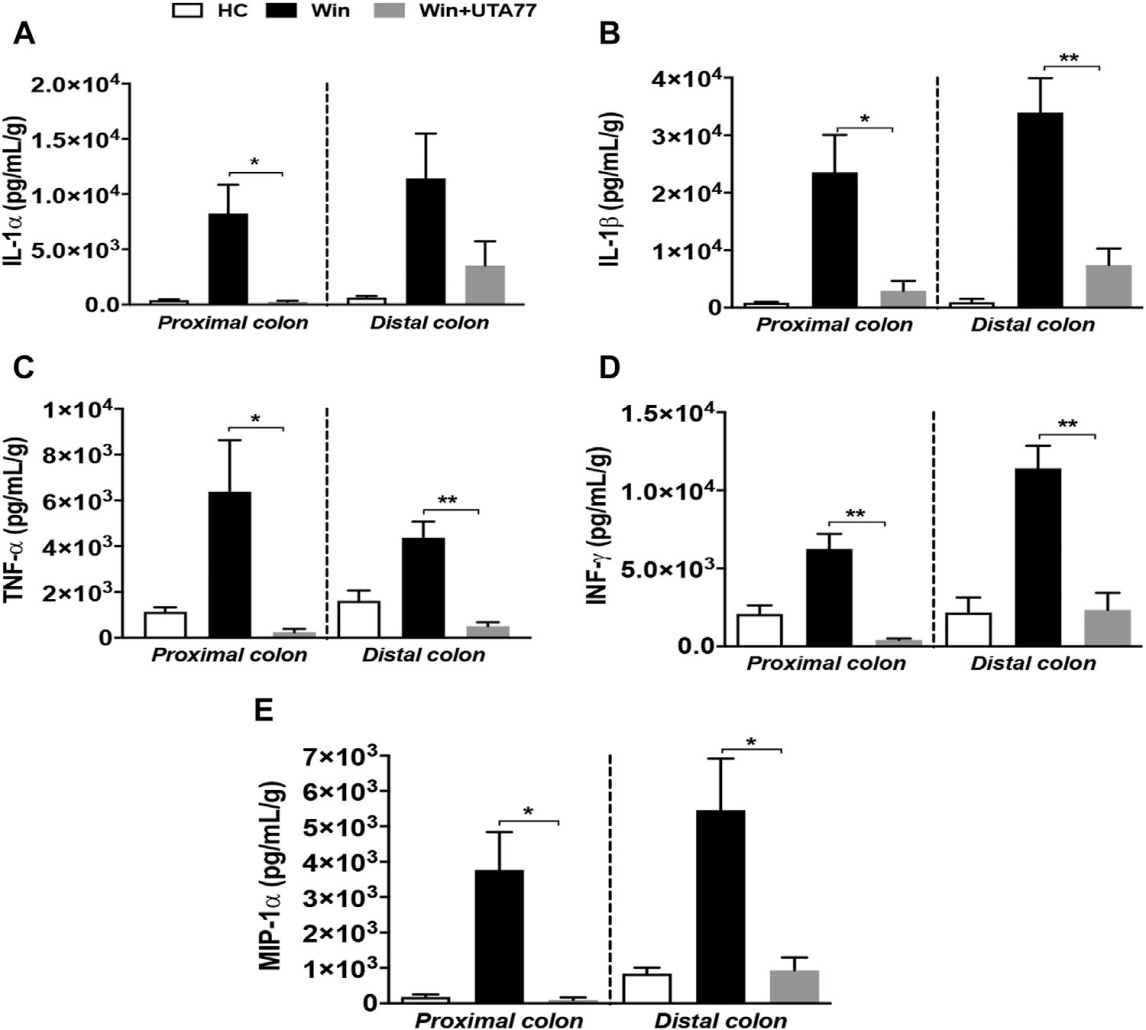
Effect of UTA77 on the level of proinflammatory cytokines and chemokines in colonic tissue explants of Winnie mice. Tissue levels of **(A)** IL-1α, **(B)** IL-1β, **(C)** TNF-α, **(D)** INF-γ, and **(E)** MIP-1α in PC and DC were quantified by Bio-Plex assay. Data expressed as mean ± SEM (*n* = 3/group) and statistical significance evaluated by one-way ANOVA followed by Tukey’s posttest where *p < 0.05 and **p < 0.01.

Similar to the acute colitis model, the immunomodulatory effect of UTA77 was examined in the Winnie mouse model. Multiple proinflammatory cytokines such as IL-1α, IL-1β, TNF-α, INF-γ, and MIP-1α (macrophage inflammatory protein one alpha) were detected in the PC and DC of untreated Winnie mice. UTA77 effectively normalised these to the levels of the HC group (Figure 10). TNF-α (Figure 10C) was strikingly reduced by UTA77 treatment in DC (*p < 0.01*) and in PC (*p* < 0.05), while IL-1α (Figure 10A) was significantly suppressed in PC but not in DC. IL-1β (Figure 10B) was also remarkably suppressed in both UTA77-treated PC (*p < 0.05*) and DC (*p < 0.01*). In addition, INF-γ (Figure 10D) was very significantly reduced in both PC and DC (*p < 0.01*) by UTA77. Furthermore, UTA77-treated Winnie also showed decreased levels of MIP-1α (Figure 10E) in both PC and DC (*p < 0.05*). No significant effects by UTA77 on several other cytokines were observed (Supplementary Figure S2).

### UTA77 Inhibits Lipid Peroxidation Only in Acute Colitis

**FIGURE 11:**
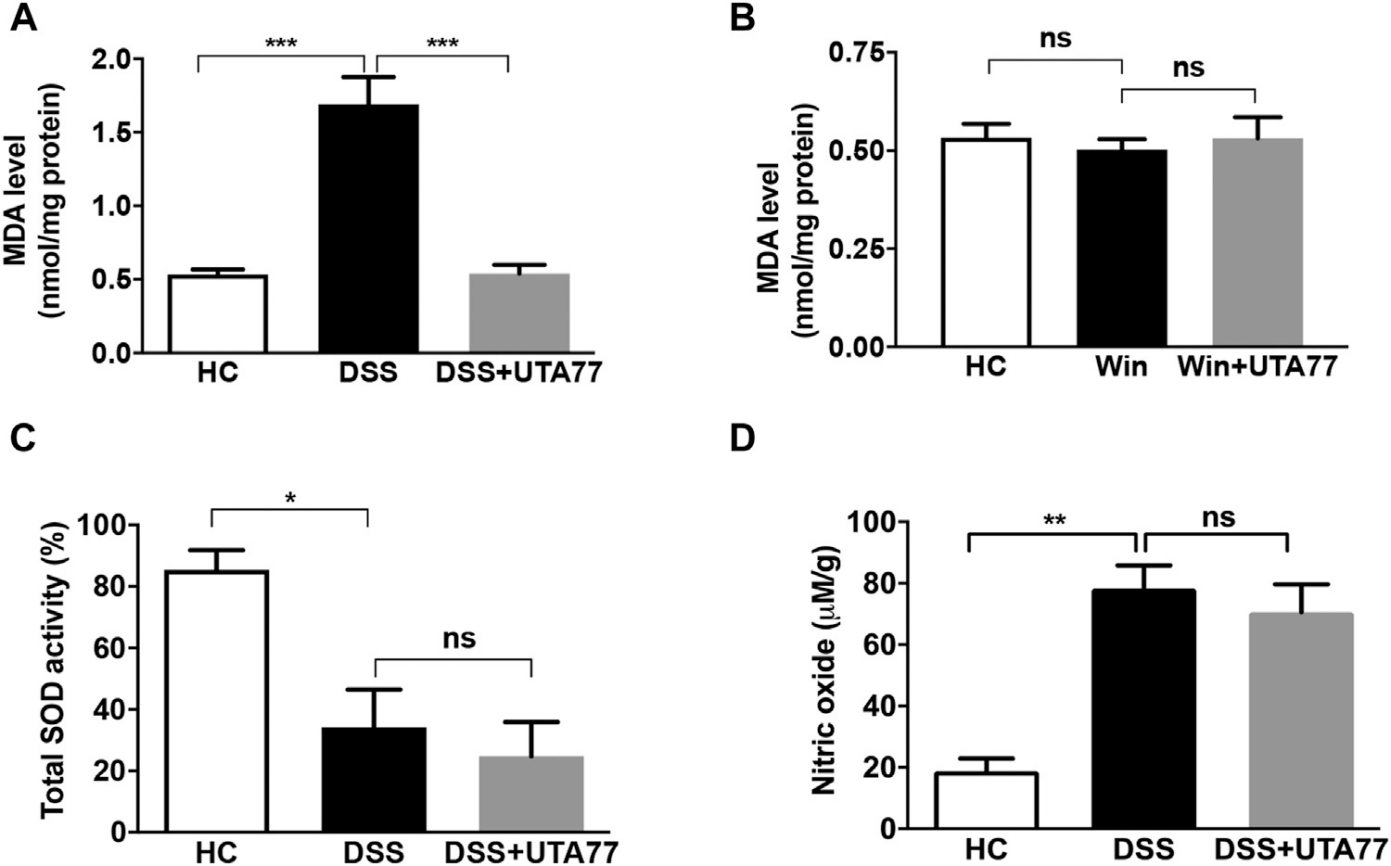
Effect of UTA77 on oxidative stress markers in acute and chronic colitis. **(A)** Malondialdehyde (MDA) levels in distal colon of acute colitic mice, **(B)** MDA level in distal colon of Winnie mice, **(C)** total SOD activity in distal colon of acute colitic mice, and **(D)** nitric oxide (NO) levels in distal colon of acute colitic mice. Data expressed as mean ± SEM (*n* = 3/group) using one-way ANOVA followed by Tukey’s posttest, where *p < 0.05, **p < 0.01, ***p < 0.001, and ns: nonsignificant.

We also assessed the antioxidant activity of UTA77 in the colon (Figure 11). While DSS administration significantly increased malondialdehyde (MDA) content in the DC of the untreated DSS group compared to the HC group (*p < 0.001*), UTA77 treatment significantly reduced MDA levels in the UTA77-treated DSS group (*p < 0.001*) (Figure 10A). Based on the results from the model of acute colitis, the potential antioxidant activity of UTA77 was also assessed in the model of chronic colitis (Figure 11B). In contrast to the model of acute colitis, no increased levels of lipid peroxidation (MDA) were detected in untreated Winnie mice compared to the HC group. Therefore, no inhibitory effect on MDA by UTA77 could be observed in the UTA77-treated Winnie group (Figure 11B).

Later on, to characterise a possible mechanism of action of UTA77 in DSS-induced acute colitis, we also assessed the and activity of the antioxidant enzyme superoxide dismutase (SOD) (Figure 11C) and nitric oxide (NO) levels in the colon tissue (Figure 11D). The SOD activity and NO levels were unaltered between the untreated DSS-control group and the UTA77-treated DSS group.

## Discussion

For the first time, the current study demonstrates the effectiveness of the novel naphthoquinone UTA77 in treatment of intestinal inflammation in models of acute and chronic experimental colitis. The results of the current study clearly demonstrate decreased severity of colitis associated with a modulated immune response, increased barrier integrity, improved tissue integrity, and reduced ER stress as a consequence of treatment with UTA77.

The novel synthetic UTA77 naphthoquinone was previously described as a potent compound to protect against mitochondrial dysfunction (Woolley et al., 2019; Feng et al., 2020b). Similar to some naturally derived naphthoquinones that were reported to ameliorate disease severity in models of colitis (Andújar et al., 2012; Fan et al., 2013; Sun et al., 2019), UTA77 successfully improved body weight loss, DAI, and colon length in acute colitis, while in chronic colitis, UTA77 improved DAI and colon length but not body weight. This selectivity to affect only some pathologies of the model mirrors an earlier study where the NLRP3 inhibitor MCC950 did not affect body weight but significantly suppressed DAI and colon length shortening in the Winnie model (Perera et al., 2018). The reason for this discrepancy could be attributed to the more severe damage to colon tissue in Winnie mice compared to the acute colitis model or that the dose used in the Winnie model was not optimised. Since UTA77 effectively alleviated histological alterations seen in both acute and chronic colitis in this study, it suggests that the UTA77 dose used in these models was effective. However, it cannot be excluded that lower doses might have resulted in similar efficacy. It has to be acknowledged that this study was not intended as a dose-finding study and hence, future studies will have to address this knowledge gap. It also opens the possibility that dose optimisation might lead to different results in different models.

Intestinal barrier dysfunction is thought to be an initial event in the propagation of UC. Loss of barrier integrity alters the expression of TJ proteins of intestinal epithelium. As a consequence, altered expression of TJ proteins increases intestinal permeability that allows the entry of harmful bacteria-derived molecules and luminal toxins into the mucosa, resulting in an uncontrolled inflammatory cascade (Turner, 2009; Holmberg et al., 2018). Overactivation of proinflammatory signalling further reduces barrier integrity and propagates this vicious cycle (Strober and Fuss, 2011). Under physiological conditions, intestinal structural integrity is maintained by the mucosal layer, composed of mucin, which protects and lubricates the intestinal tract (Tytgat et al., 1996). Previous studies reported altered expression or loss of TJ proteins occludin and ZO-1 and reduced mucin expression in colitis patients and in mouse models of intestinal inflammation (Kucharzik et al., 2001; Poritz et al., 2007; Turner, 2009; Poritz et al., 2011; Das et al., 2012; Li et al., 2014; Nighot et al., 2015), which the present study confirmed. Since UTA77 dramatically alleviated these pathologies, this could indicate that the protective effect of UTA77 might be associated with the protection of mucosal barrier integrity that paralleled the protection against proinflammatory cytokine release. However, the present study did not assess whether UTA77 directly increased the expression of TJ proteins or protected against their DSS-mediated degradation, which will be the topic of future studies.

During inflammation, oxidative stress arises due to overproduction of ROS and weakening of antioxidant defence machinery (Tian et al., 2017). Increased levels of ROS in intestinal epithelium damage colonic mucosal tissues by mediating lipid peroxidation and free radicals (Siegel et al., 2004; Tian et al., 2017). Previously, naphthoquinones also protected against oxidative stress in preclinical models (Vos et al., 2012; Josey et al., 2013; Rahn et al., 2014). In this study, treatment with UTA77 inhibited lipid peroxidation by lowering MDA levels only under conditions of acute colitis. Under conditions of chronic colitis in Winnie mice, MDA levels were indistinguishable between all the three groups, indicating a lack of ROS accumulation in young Winnie mice. Our data therefore suggest that oxidative stress might not be causative or act as a central event in the development of colitis but instead represents an additional pathological factor along with the major components of intestinal inflammation such as barrier dysfunction, cytokine dysregulation, and microbial dysbiosis. Furthermore, UTA77 was unable to alter the SOD activity and the levels of NO in DSS-induced mice which suggest that UTA77 adopted another mode of action in ameliorating colitis.

The transcription factor CHOP is highly expressed during the dysregulation of ER and mitochondrial function (Zhao et al., 2002; Zhang and Kaufman, 2008). CHOP is strongly implicated in the development of colitis as it is upregulated in response to trinitrobenzene sulphonic acid (TNBS) or DSS administration, while CHOP-deficient mice resist the development of colitis (Namba et al., 2009). Indeed, increased expression of CHOP and CHOP-mediated apoptosis was reported in Winnie mouse previously, where intestinal inflammation arises as a direct consequence of aberrant ER stress (Heazlewood et al., 2008; Das et al., 2013; Kunde et al., 2017). UTA77 markedly reduced CHOP expression in Winnie mice and therefore may have ameliorated inflammation by reducing ER stress and cell death of epithelial cells. Although a single study reported an increase in CHOP expression in response to the natural naphthoquinone plumbagin in the treatment of prostate cancer (Huang et al., 2018), this discrepancy could be due to the different pathophysiology of tissue and disease and the very different chemical structure of the naphthoquinone used in this study compared to UTA77. Given the close steric and functional connectivity between mitochondria and ER, the presence of mitochondrial dysfunction and ER stress in IBD does not come as a surprise, with increased CHOP levels associated with the unfolded protein responses in both mitochondria and ER (Horibe and Hoogenraad, 2007; Rath et al., 2012). Therefore, the mitoprotective activity of UTA77 might have preferentially targeted mitochondrial dysfunction and downregulated CHOP expression, the protective effect of which could have extended to the ER. Extended studies will be required to explore the association of mitochondrial and ER stress in Winnie and how this is regulated by CHOP to initiate chronic UC.

Defective barrier integrity and oxidative and ER stress mediate colonic inflammation by increased release of proinflammatory mediators from immune cells and mitochondria (Kaser and Blumberg, 2009; Roediger, 1980; Heazlewood et al., 2008; Guo et al., 2015; Novak and Mollen, 2015). Anti-inflammatory activity of natural naphthoquinones was previously reported in LPS-induced neuroinflammation in BV-2 microglial cells, in the DSS colitis model of acute colitis, and in LPS-induced inflammation in mice. This anti-inflammatory activity involved the downregulation of proinflammatory cytokines such as IL-1β, TNF-α, INF- γ, IL-1α, IL-6, G-CSF, and IL-12p70 cytokines (Sun et al., 2019; Andújar et al., 2012; Lomba et al., 2017; Messeha et al., 2017). The present study confirmed and extended this effect by detecting a substantial reduction of IL-1α, IL-1β, IL-6, TNF-α, INF-γ, G-CSF, IL-13, GM-CSF, IL-3, MIP-1α, RANTES, and eotaxin cytokines in response to UTA77 treatment in both colitis models. Although this confirms the general anti-inflammatory property of some naphthoquinones and UTA77, some cytokines remained unaffected by UTA77 administration (Supplementary Figure S1, S2). While it is possible that suboptimal dosing of UTA77 restricted the suppression of these cytokines, it may also be possible that the specific mode of action of UTA77 only affects some signalling pathways. Among the cytokines inhibited by UTA77, IL-1β and TNF-α were specifically downregulated in the distal colon. Both cytokines are produced by proinflammatory M1 macrophages at the mucosal surfaces. Considering the amount of IL-1β secreted in the distal colon of Winnie mice together with our earlier results that a small molecule inhibitor of the NLRP3 inflammasome ameliorates colitis in Winnie mice (Perera et al., 2018), it is possible that UTA77 also prevents NLRP3 inflammasome activation. Future studies will investigate if inflammasome activation is affected by UTA77 to assess this possibility.

Interestingly, IL-10, which is well known for its anti-inflammatory response, was elevated by DSS induction in the present and previous studies as well as in UC patients (Melgar et al., 2003; Shastri et al., 2020a). Since increased IL-10 levels could represent a compensatory mechanism to reduce colon inflammation, the overall reduction of proinflammatory cytokines by UTA77 could have reduced the elevated level of IL-10 as a compensatory mechanism.

Although, there is no clinical evidence at present that would support the therapeutic potential of naphthoquinones in human UC, encouraging data with some natural naphthoquinones have been reported in mouse models of colitis (Andújar et al., 2012; Fan et al., 2013; Pile et al., 2013; Sun et al., 2019). These studies typically only focused on some limited parameters of disease pathology utilising DSS or TNBS mouse models of colitis. In contrast, as an extension, the present study demonstrated protection by UTA77 with regard to increased intestinal barrier integrity, mucus secretion and reduced ER stress, and lipid peroxidation. This suggests that pharmaceuticals that target all these factors simultaneously may be more effectively reducing the severity of intestinal inflammation than drugs that target only a single aspect of the pathology. In this context, UTA77 may achieve this by protecting mitochondrial function and bioenergetics, although the current study did not identify the direct molecular target; that is responsible for the observed protective activity. Future studies will address the potential role of UTA77 in intestinal mucosal healing by specifically addressing the role of UTA77 in the nexus of ER-mitochondria interactions.

UTA77 was identified as mitoprotective, while other naphthoquinones in the same group of compounds did not share this activity (Woolley et al., 2019). While the redox activity of quinone moiety is essential for the activity of the molecule, it is not sufficient for this activity. In fact, it was demonstrated that the composition of side chain provides the molecule with specific activity (Woolley et al., 2019). This suggests that the quinone as an active moiety interacts with its target by the characteristic composition of the side chain. However, the exact molecular target remains to be identified by future studies.

Overall, our study provided vital information towards understanding the therapeutic role of the novel mitoprotective UTA77 naphthoquinone in colitis. Due to its low toxicity, metabolic stability, and therapeutic efficacy in both acute and chronic models of colitis, UTA77 may also be considered as a promising drug candidate to mitigate intestinal inflammation in human UC.

## Data Availability

The original contributions presented in the study are included in the article/Supplementary Material; further inquiries can be directed to the corresponding authors.
