# Supplementary material for: Short-Chain Naphthoquinone Protects Against Both Acute and Spontaneous Chronic Murine Colitis by Alleviating Inflammatory Responses
Source: Front Pharmacol. 2021 Aug 23;12:709973. doi: 10.3389/fphar.2021.709973 (PMC8419285; doi:10.3389/fphar.2021.709973)
Supplement: Supplementary file 1 [file DataSheet1.ZIP › Supplementary Figure 1.pdf]

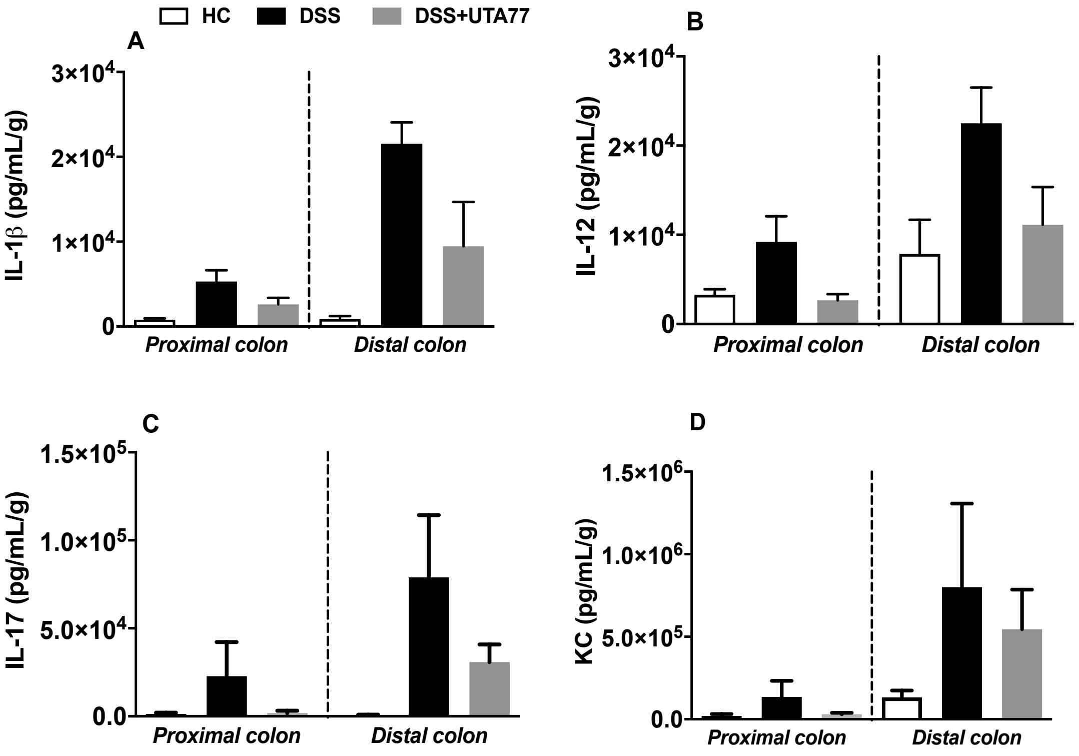

Supplementary Figure 1. Effect of UTA77 on the levels of inflammatory cytokines in colon tissue of DSS-induced acute colitic mice. **(A)** IL-1β, **(B)** IL-12, **(C)** IL-17 and **(D)** KC in proximal and distal colon were quantified by Bio-Plex assay. Data expressed as mean ± SEM (n=3/group). Statistical significance evaluated by One-way ANOVA followed by Tukey's post test.
