# Supplementary figures and images for: Short-Chain Naphthoquinone Protects Against Both Acute and Spontaneous Chronic Murine Colitis by Alleviating Inflammatory Responses
Source: Front Pharmacol. 2021 Aug 23;12:709973. doi: 10.3389/fphar.2021.709973 (PMC8419285; doi:10.3389/fphar.2021.709973)

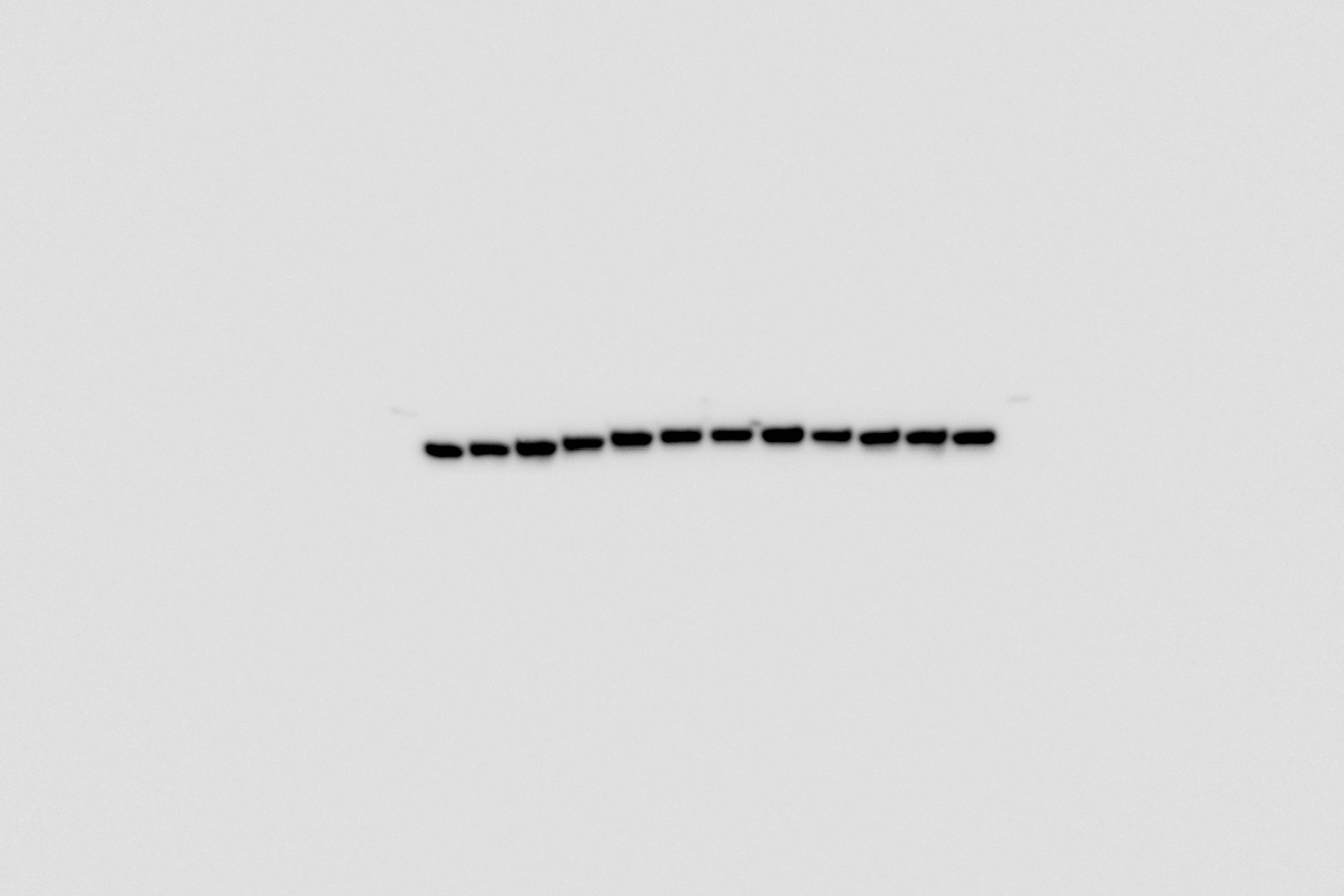

Supplement: Supplementary file 2 [file DataSheet2.ZIP › Beta-Actin.tif]

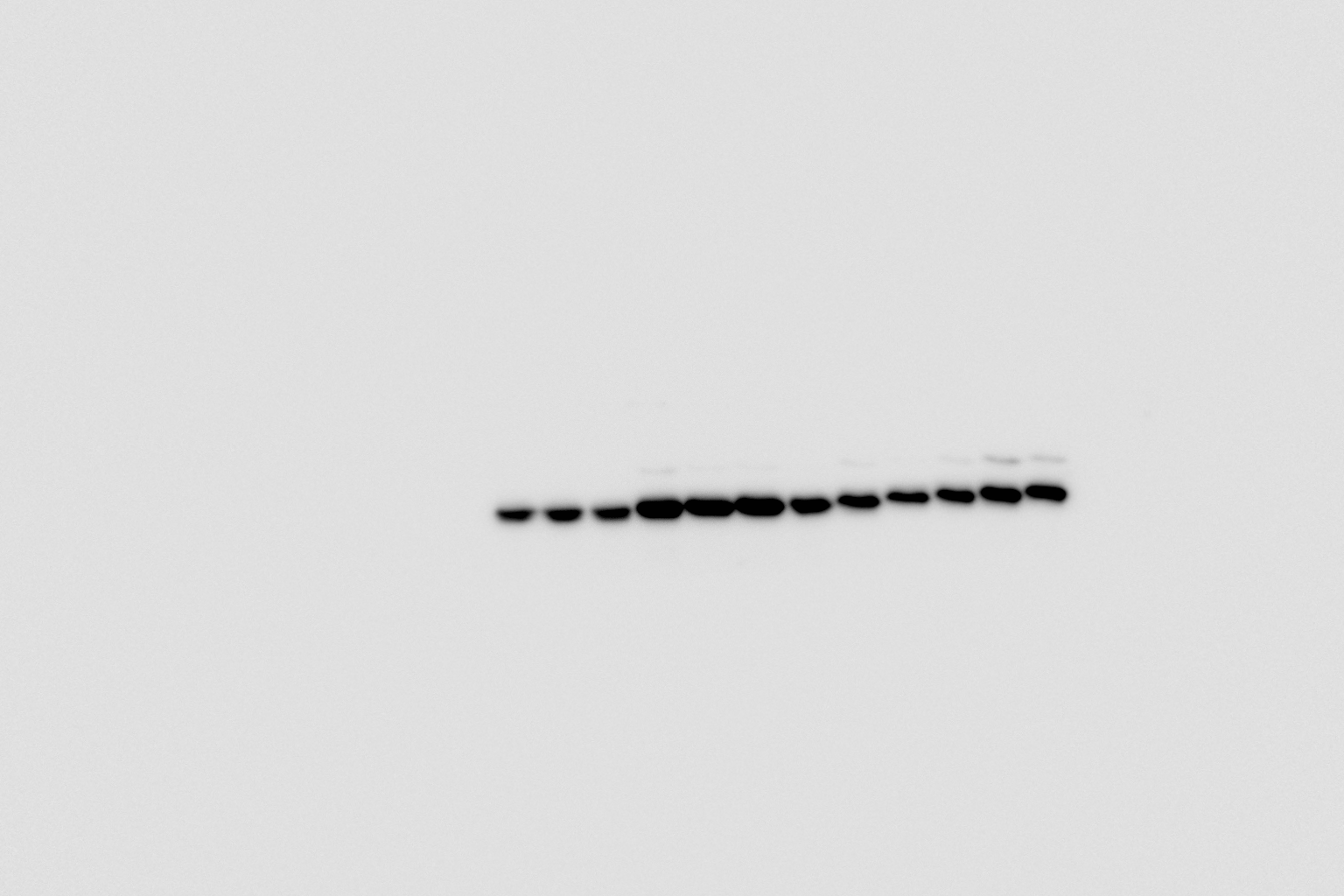

Supplement: Supplementary file 2 [file DataSheet2.ZIP › CHOP.tif]

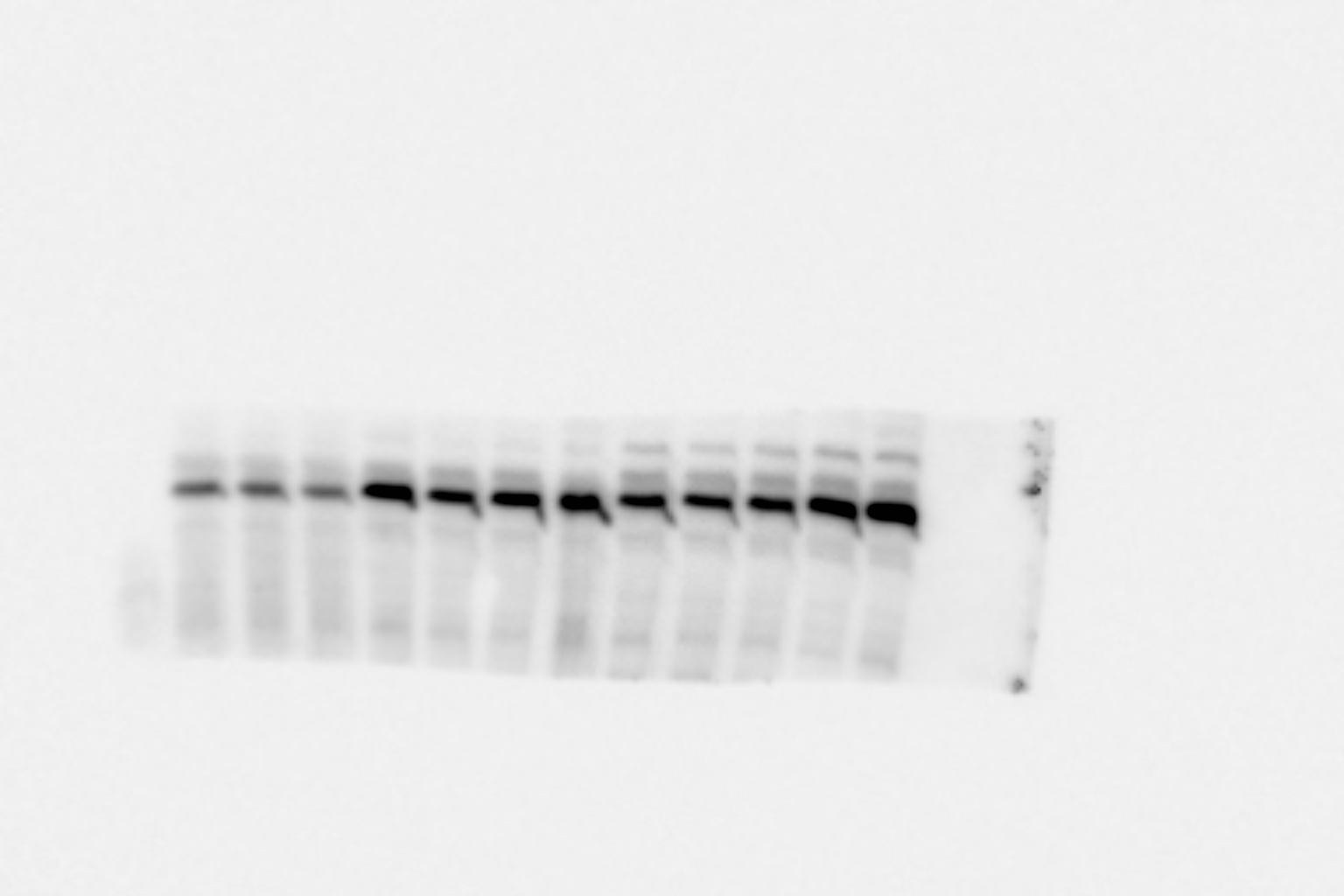

Supplement: Supplementary file 2 [file DataSheet2.ZIP › GRP78.tif]

## GRP78

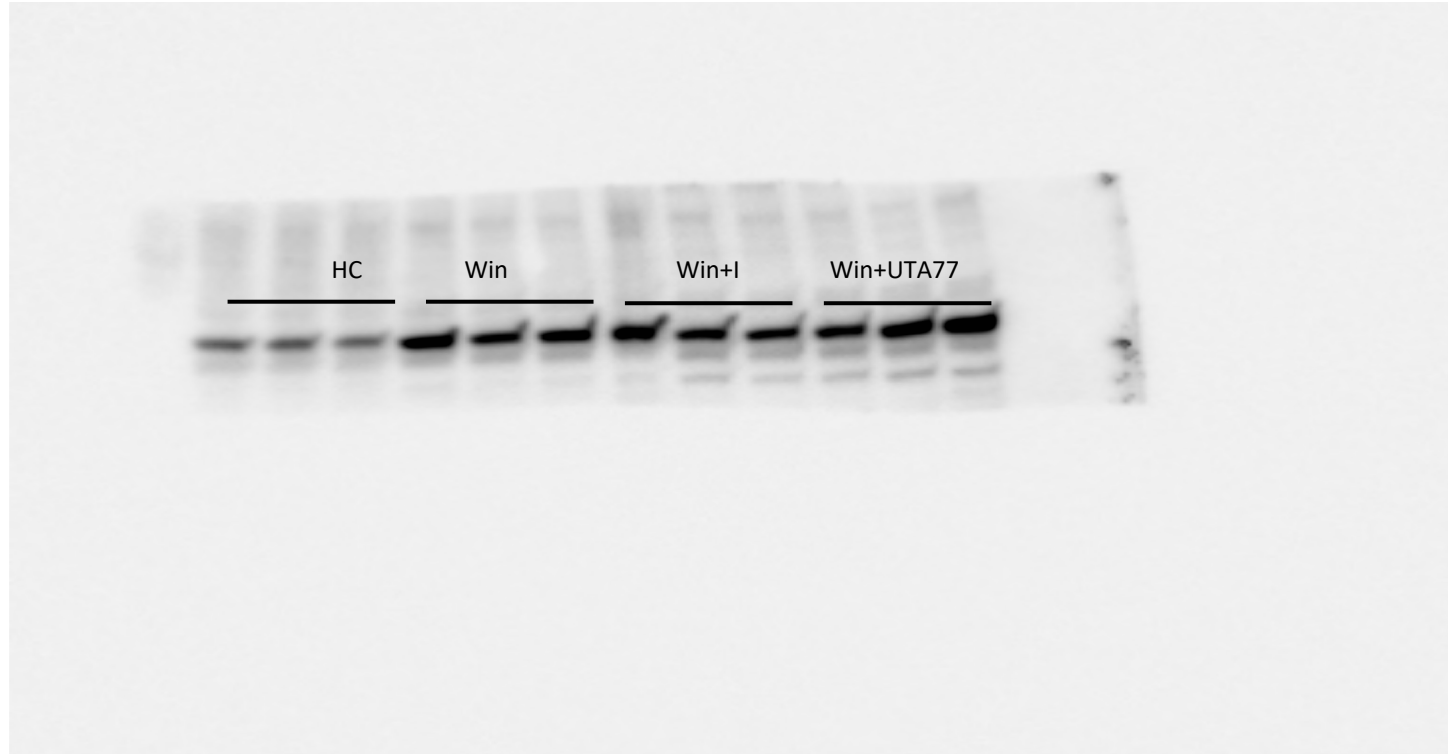

**Win + I = Another agent (idebenone)**

## CHOP

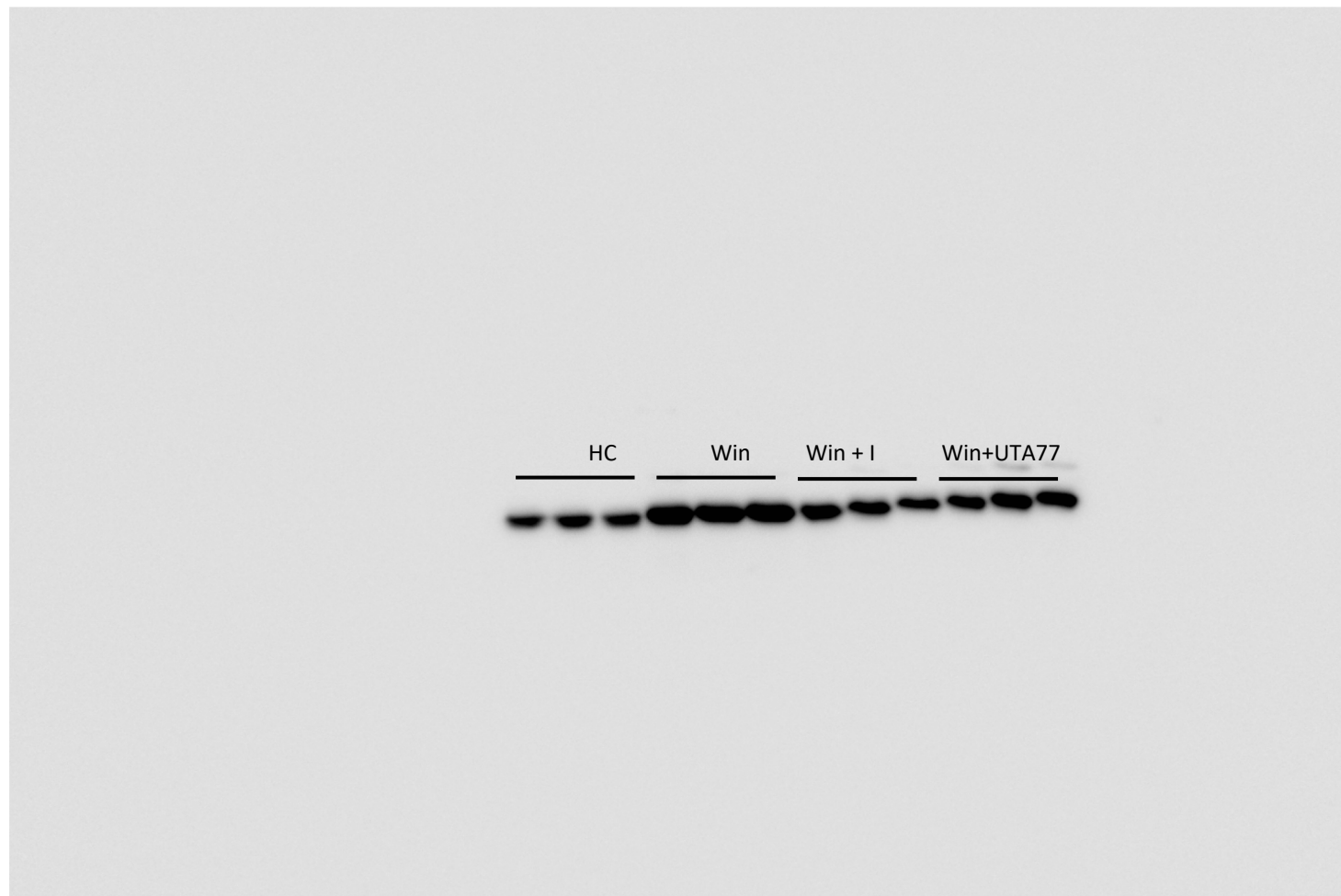

## Beta-actin

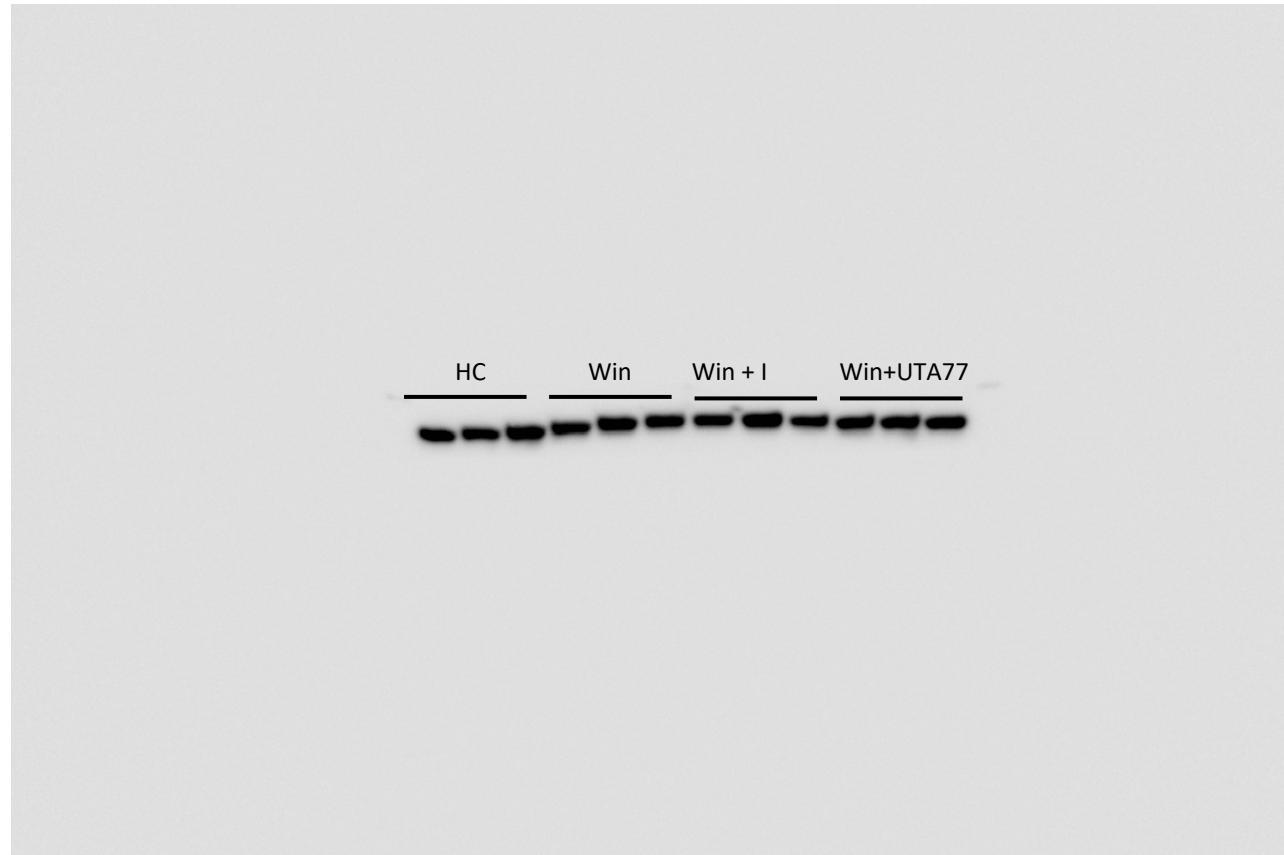

Supplement: Supplementary file 2 [file DataSheet2.ZIP › Western blot gel .pdf]
